# Genome-wide CRISPR knockout screens identify NCAPG as an essential oncogene for hepatocellular carcinoma tumor growth

**DOI:** 10.1096/fj.201802213RR

**Published:** 2019-04-25

**Authors:** Yu Wang, Bin Gao, Peng Yang Tan, Yohana Ayupriyanti Handoko, Karthik Sekar, Amudha Deivasigamani, Veerabrahma Pratap Seshachalam, Han-Yue OuYang, Ming Shi, Chan Xie, Brian Kim Poh Goh, London Lucien Ooi, Kam Man Hui

**Affiliations:** * Division of Cellular and Molecular Research, National Cancer Centre Singapore, Singapore;; † Department of Hepatobiliary Oncology, Sun Yat-Sen University Cancer Center, Guangzhou, China;; ‡ Department of Infectious Diseases, The Third Affiliated Hospital of Sun Yat-Sen University, Guangzhou, China;; § Department of Hepato-Pancreato-Biliary and Transplant Surgery, Singapore General Hospital, Singapore;; ¶ Division of Surgical Oncology, National Cancer Centre Singapore, Singapore;; ‖ Department of Biochemistry, Yong Loo Lin School of Medicine, National University of Singapore (NUS), Singapore;; # Institute of Molecular and Cell Biology, Agency for Science, Technology, and Research (A*STAR), Singapore;; ** Duke-NUS Medical School, Singapore

**Keywords:** cell cycle, therapeutic target, tumor recurrence

## Abstract

Hepatocellular carcinoma (HCC) is a common and deadly cancer with limited treatment options. Through genome-wide growth depletion screens using clustered regularly interspaced short palindromic repeats and expression profiling of primary HCC tumors, we identified 13 clinically relevant target genes with therapeutic potential. Subsequent functional annotation analysis revealed significant enrichment of these 13 genes in the cell cycle, cell death, and survival pathways. Non–structural maintenance of chromosomes condensin I complex subunit G (NCAPG) was ranked the highest among the depletion screens and multiple HCC expression datasets. Transient inhibition of NCAPG using specific small interfering RNAs resulted in a significant reduction in cell growth, migration, and the down-regulation of mitochondrial gene expression *in vitro*. Small homologous RNA–mediated knockdown of NCAPG significantly impaired cell viability, caused aberrant mitotic division, fragmented the mitochondrial network, and increased cell death *in vitro*. HCC cells with a reduced expression of NCAPG formed significantly smaller xenograft tumors *in vivo*. Importantly, high NCAPG expression was significantly associated with poorer overall and disease-free survival in HCC patients. High NCAPG expression is a novel prognostic biomarker to predict HCC early recurrence after surgical resection. In conclusion, NCAPG is an essential gene for HCC tumor cell survival. It represents a promising novel target for treating HCC and a prognostic biomarker for clinical management of HCC.—Wang, Y., Gao, B., Tan, P. Y., Handoko, Y. A., Sekar, K., Deivasigamani, A., Seshachalam, V. P., OuYang, H.-Y., Shi, M., Xie, C., Goh, B. K. P., Ooi, L. L., Hui, K. M. Genome-wide CRISPR knockout screens identify NCAPG as an essential oncogene for hepatocellular carcinoma tumor growth.

Hepatocellular carcinoma (HCC) accounts for more than 85% of all primary liver cancer and is ranked as the sixth most common and second leading cause of cancer-related deaths worldwide (1–3). Surgical resection remains the only common treatment modality to consistently confer survival benefits to the approximately 15% of HCC patients who have their tumor detected early. In contrast, conventional radio- and chemotherapies are largely ineffective (4–6). Sorafenib is the only Food and Drug Administration–approved systemic chemotherapy drug for advanced-stage HCC, and it suffers from a low response rate and high number of side effects, in addition to being a very costly drug (7, 8). Despite much effort in developing new therapeutic targets, the next breakthrough remains elusive (9). Therefore, it is an urgent, unmet clinical need to find novel targets to enable the development of effective molecularly targeted therapy for HCC.

Thousands of clinical HCC samples have been examined to search for suitable therapeutic targets using various genome-wide technologies, such as microarrays and, more recently, next-generation sequencing platforms. Unfortunately, no single sequence of molecular or genetic alterations contributes to the development of HCC (3, 10). Tumor protein P53 is the most frequently mutated tumor suppressor gene, whereas Wingless/Integrated–β-catenin and Janus kinase–signal transducer and activator of transcription pathways were commonly altered in HCC (11). In contrast, high cell proliferation was the most important and consistent factor significantly associated with poorly differentiated tumors, tumor recurrence, and poorer survival of the patients (11, 12). Taken together, the known cancer-associated genetic or epigenetic aberrations in the upstream pathways will eventually, in one way or another, exert their influence to alter the cell cycle. After all, aberrant cell growth is the definitive hallmark of cancer. Conventional chemotherapy agents targeting the rapidly dividing cells are still the most effective treatment options for many cancers today (13). Therefore, in the absence of clear genetic alterations at the genomic DNA level, a molecular target that is specifically required by HCC tumor cells for their aberrant cell cycle process may represent a novel approach for targeted treatment of HCC.

In this study, we performed genome-wide clustered regularly interspaced short palindromic repeats (CRISPR) depletion screens to identify cellular targets that are essential for HCC tumor growth. Non–structural maintenance of chromosomes condensin I complex subunit G (NCAPG) was a clinically relevant target because it has a tumor-specific up-regulation in HCC. NCAPG is further characterized to be essential for tumor cell growth and therefore is a promising novel therapeutic target to treat HCC.

## MATERIALS AND METHODS

### Patient samples and cell lines

Resected HCC tumor samples and matched histologically normal samples were obtained from Singapore General Hospital and the Department of Hepatobiliary Oncology, Sun Yat-Sen University Cancer Center, Guangzhou, China. All samples were collected in accordance with the protocols approved by the respective institutional review boards, and informed consent was obtained from all subjects before sample collection. All resected HCC tumors were confirmed through histopathology.

The HepG2 and SNU449 cells were purchased from American Type Culture Collection (Manassas, VA, USA) (HB-8065) with proof of authenticity. HCCLM3 was a kind gift from Prof. Zhao-You Tang at the Liver Cancer Institute (Zhongshan Hospital–Fudan University, Shanghai, China) (14), and it was authenticated using the short tandem repeat profiling authentication test by Bio-Synthesis (Lewisville, TX, USA). Huh7 cells were bought from the Japanese Collection of Research Bioresources Cell Bank (Osaka, Japan) and werere validated by Genetica DNA Laboratories (Cincinnati, OH, USA). All HCC cell lines were grown in DMEM with 4.5 g/L high glucose, 15 mM HEPES buffer, and 10% fetal calf serum. Human primary hepatocytes were purchased from Beckton Dickinson (Franklin Lakes, NJ, USA) and grown in Hepatocyte Maintenance Medium (Lonza, Basel, Switerland) with 5% fetal calf serum. All cells were cultured in a humidified incubator at 37°C with 5% CO_2_. Human hepatocytes and HepG2 cells were grown on collagen-coated surfaces to enable monolayer growth.

### Genome-wide CRISPR knockout growth screen in human HCC cells

The human Genome-Scale CRISPR Knock-Out (GeCKO) v2 CRISPR knockout pooled library was a gift from Feng Zhang (Broad Institute, Massachusetts Institute of Technology, Cambridge, MA, USA) (Addgene 1000000048). The pooled library contained 6 redundant guide RNAs (gRNAs) targeting each of the 19,050 coding genes. HCCLM3 and SNU449 cells were transduced with the lentiviral particles containing a genome-wide set of gRNAs at a multiplicity of infection (MOI) of 0.2 to ensure a single-copy integration per cell in >95% of the population and over 100 times coverage of the entire library. The transduced cells were selected with 4 µg/ml puromycin for stable integration. The resultant GeCKO-ready cells were harvested at the start (time zero) and at d 30 (end point), when cells had completed ∼10 population doublings under normal growth conditions. Genomic DNA was extracted using an E.Z.N.A. HP Tissue DNA Maxi Kit (Omega Bio-tek, Norcross, GA, USA), and the integrated gRNA region was PCR amplified using a Q5 High-Fidelity DNA Polymerase (New England Biolabs, Ipswich, MA, USA) with V2 adaptor primers. Illumina-compatible libraries were generated using universal forward primers and sample-specific barcoded reverse primers. The libraries were pooled and sequenced on an Illumina HiSeq SE100 platform (Illumina, San Diego, CA, USA). We used the Model-based Analysis of Genome-wide CRISPR/Cas9 Knockout (MAGeCK) algorithm to identify the top hits and ranked them according to their degrees of depletion in the end point sample (15). Primer sequences used in this screen are provided in Supplemental Table S1. The CRISPR screening data were deposited at the Gene Expression Omnibus repository with accession number GSE94509.

### Whole-genome expression microarray analysis

Total RNA from 70 HCC tumors and 37 matched histologically normal tissues were extracted using Trizol Reagent (Thermo Fisher Scientific, Waltham, MA, USA) according to the manufacturer’s protocol. Five micrograms of purified total RNA was reverse transcribed, labeled with biotin, and hybridized onto the GeneChip Human Genome U133 Plus 2.0 Array (Thermo Fisher Scientific) to determine the expression level of each gene as previously described (16). Raw expression data were normalized using GeneChip Robust Multiarray Averaging method (Partek Genomics Suite v.6.6 software; Partek, St. Louis, MO, USA). ANOVA with false discovery rate (FDR) multiple-test correction was used to select significantly up-regulated genes between tumor samples and matched normal samples using fold change (FC) values >2 and an FDR *q* value <0.05. The microarray data have been deposited in the ArrayExpress public database with accession numbers E-MEXP-84 and E-TABM-292.

### Knockdown of NCAPG in HCC cell lines using small interfering RNAs or small homologous RNAs

Small interfering RNAs (siRNAs) targeting NCAPG or nontargeting control siRNAs were introduced into cells at 20 µM concentration through either electroporation using a BTX electroporator at 180 V and 100 ms (BTX, San Diego, CA, USA) or siPORT Amine reagents (Thermo Fisher Scientific) following the manufacturer’s protocol. Small homologous RNAs (shRNAs) targeting NCAPG or nontargeting controls were cloned into constitutive pLKO.1 Puro (Addgene 8453) or tetracycline-inducible tet-pLKO.1 Puro constructs (Addgene 21915) according to the manufacturer’s protocol. HCC cells were infected with the respective viruses at an MOI of 0.2 in the presence of 2 μg/ml polybrene and selected using 2 or 4 µg/ml puromycin. The stable cells were maintained in culture with 2 or 4 µg/ml puromycin to ensure stable integration. One microgram per milliliter doxycycline (dox) was used to activate the shRNA expression in Tet-on–inducible constructs. Down-regulation of NCAPG was confirmed using quantitative RT-PCR (qRT-PCR) and Western blot analysis. Sequences or the catalog numbers of the siRNAs and shRNAs used are provided in Supplemental Table S1.

### Measurement of cell growth and migration

The CellTiter 96 AQueous One Solution Cell Proliferation Assay (MTS) was used to measure the cell proliferation potential of cells transfected with siRNAs targeting NCAPG or control siRNAs, following the manufacturer’ protocol (Promega, Madison, WI, USA). The cell viability testing with the Trypan blue exclusion method using the Vi-Cell XR cell counter (Beckman Coulter, Brea, CA, USA) was used to determine the exact cell numbers transfected with siRNAs targeting NCAPG or control siRNAs over time. The IncuCyte ZOOM Continuous Live-cell Imaging and Analysis System (Essen BioScience, Ann Arbor, MI, USA) was employed to monitor the growth of cells with stable knockdown of NCAPG or control. Growth measurements were recorded as relative confluence over time.

HCC cell migration was measured using a wound healing assay. HCC cells were seeded into 96-well image lock plates. A fixed-width wound was made to 100% confluent cells using the semimanual WoundMaker tool (Essen Bioscience). The plates were imaged hourly for 24 h. The wound healing was measured as a relative cell confluency inside the wound region over time using the IncuCyte software (Essen Bioscience).

### RNA sequencing

HepG2 cells were transfected with 2 independent control siRNAs (siControl-1 and -2) against 4 independent siRNAs targeting NCAPG (siNCAPG-1, -2, -3, and -4). The total RNA was extracted using RNAzol 48 h posttransfection, and paired-end libraries were prepared using the Illumina TruSeq v2 Sample Prep Kit (15596-026; Illumina, San Diego, CA, USA), starting with 1 μg total RNA. Libraries were sequenced on an Illumina HiSeq 2000. RNA sequencing (RNA-seq) data analysis was performed with Partek Flow v.5.0.16.0708 (Partek). The raw paired-end reads were aligned using TopHat v.2-2.1.0 (Johns Hopkins University Center for Computational Biology, Baltimore, MD, USA) against the Hg19 reference genome. Gencode v.19 (*https://www.gencodegenes.org/human/release_19.html#*) annotation was used to quantify the aligned reads using the Partek expectation–maximization method. Differential expression analysis was performed using Partek’s gene-specific analysis; values of *P* < 0.01 and absolute FC >1.5 were used as cutoffs to select the differentially expressed genes for Gene Ontology analysis.

### Xenograft tumor growth *in vivo*

All mice experiments were performed in accordance with Animal Research: Reporting of *In Vivo* Experiments (ARRIVE) standards and approved by the SingHealth Institutional Animal Care and Use Committee. Huh7 cells stably expressing tetracycline-inducible shRNA targeting NCAPG (tet-pLKO.puro-shNCAPG-1) or scrambled control (tet-pLKO.puro-shControl-1) were transplanted subcutaneously with 3 million cells in 10% Matrigel (Corning, Corning, NY, USA) per mouse, bilaterally, with 4 mice/group. Fifty micrograms of dox was given to the Dox-On group *via* oral gavage twice a week. Tumor volume measurements were determined manually using calipers. Tumor weight was measured when mice were euthanized 47 d after tumor cell administration.

### Cell cycle analysis

Cells were arrested at the G_1_/S phase using a double thymidine protocol. Briefly, cells were initially treated with 2 mg/ml thymidine for 18 h, washed once with PBS, and released in normal medium for 4 h before arresting with 2 mg/ml thymidine for a further 18 h. The resultant cells were washed once with PBS and released from the G_1_/S phase by culturing in normal growth medium. Cells were harvested at various time points postrelease, fixed in ice-cold ethanol, and stained with 20 µg/ml propidium iodide in PBS and analyzed by fluorescence-activated cell sorting (FACS).

### Immunofluorescence staining and live-cell imaging

HCC cells that stably express H2B–enhanced green fluorescent protein and shRNA targeting NCAPG or scrambled control were fixed using 10% neutral buffered formalin and stained using antibodies against NCAPG, β-tubulin, γ-tubulin, and DAPI to visualize the localization of these proteins at various stages of mitosis. In a separate set of experiments, the above cells were imaged using the Nikon N-storm/spinning disc confocal microscope (Nikon, Tokyo, Japan) every 10 min for 24 h to monitor living cells’ progression through mitosis. MitoTracker Red CMXRos (Thermo Fisher Scientific) was used to specifically stain mitochondria, and mitochondria were visualized using a Zeiss Elyra superresolution microscope (Zeiss, Oberkochen, Germany) and structured illumination.

### Bioinformatic analysis

Kyoto Encyclopedia of Genes and Genomes (KEGG) pathway analysis was performed using the Enrichr (*http://amp.pharm.mssm.edu/Enrichr/*) algorithm (17, 18). Ingenuity pathway analysis software (Qiagen, Hilden, Germany) was used to construct cell cycle, cell death, and survival interaction networks. Pearson correlation with mean linkage was used to construct a correlation matrix using microarray data and the Partek Genomics Suite software. Kaplan-Meier survival analysis was used to analyze the association of NCAPG status with patient overall and disease-free survival in IBM SPSS (Chicago, IL, USA). An unpaired 2-tailed Student’s *t* test was performed to analyze the significance of differences between sample means obtained from 3 independent experiments. The individual predictive accuracy of the NCAPG was determined using a univariate binary logistic regression analysis. The discriminatory power of NCAPG was also shown using ROC curve plots. Multivariate binary logistic regression using a stepwise forward method was used to determine the predictive accuracy of NCAPG and liver cirrhosis in combination. Statistical significance was attained when the value of *P* < 0.05.

## RESULTS

### CRISPR growth screen identifies hits important for cell growth

**Figure 1:**
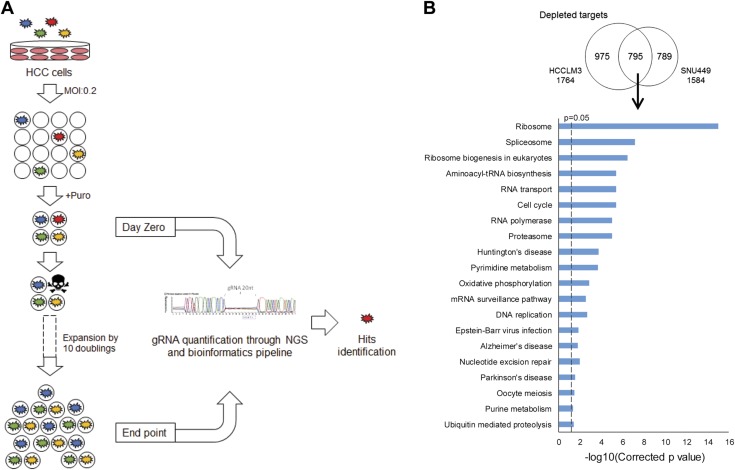
Genome-wide CRISPR depletion screen. *A*) Schema of the screen. Human HCC cell lines, HCCLM3 and SNU449, were transduced with a GeCKO v2 lentivirus gRNA library at an MOI of 0.2 and selected with puromycin (Puro) (4 µg/ml), and the resultant GeCKO-ready cells were allowed to grow for 10 doublings in normal growth conditions. Cells harboring gRNAs that knock out cellular targets that are important for cell growth and survival will have a disadvantage in proliferation and therefore result in the respective gRNAs being depleted from the final population. gRNAs from samples collected at end point are compared against those at d 0 using next-generation sequencing (NGS) to identify hits, which are defined as growth-promoting genes with multiple targeting gRNAs significantly depleted in end point samples. *B*) Hits from the CRISPR depletion screen were significantly enriched in essential cellular pathways. Top: Venn diagram showing a total of 795 hits that were commonly depleted in both HCCLM3 and SNU449 cell lines at the end of the screen. Bottom: KEGG pathway analysis showing significant enrichment in essential pathways (such as the ribosome, cell cycle, and DNA replication) among these 795 commonly depleted targets. The *x* axis shows the *P* value from a hypergeometric test adjusted by the multiple-test correction. The dashed line indicates a threshold of corrected value of *P* = 0.05.

We performed genome-wide CRISPR depletion screens using the human CRISPR knockout pooled library v2 to identify genes that are essential for tumor cell growth in HCCLM3 and SNU449 cell lines (**Fig. 1*A***) (19). We achieved a comprehensive coverage of 99.94% of gRNAs and 100% of genes represented in the library (Supplemental Table S2). If a gRNA knocks out an essential gene for HCC cell growth, the cells with that particular gRNA will proliferate less and result in the particular gRNA eventually being depleted from the final population. Using the MAGeCK algorithm to select the top hits (15), we identified a total of 795 genes that were commonly depleted in both HCCLM3 and SNU449 cells. These 795 depleted genes showed significant enrichment in 20 KEGG pathways, including ribosome, cell cycle, and DNA replication, which are crucial for cell survival and growth (Fig. 1*B* and Supplemental Table S3). In contrast, a total of 112 genes were commonly overrepresented in the final populations in both cell lines. These commonly enriched genes showed significant enrichment in mammalian target of rapamycin signaling and p53 signaling pathways, both of which are well-known negative regulators of cell growth (Supplemental Fig. S1). Taken together, our data demonstrated that the screens are highly robust to identify 795 genes that are essential for HCC tumor cell growth.

### Clinically relevant targets from the CRISPR screen are enriched in the cell cycle pathway

**Figure 2:**
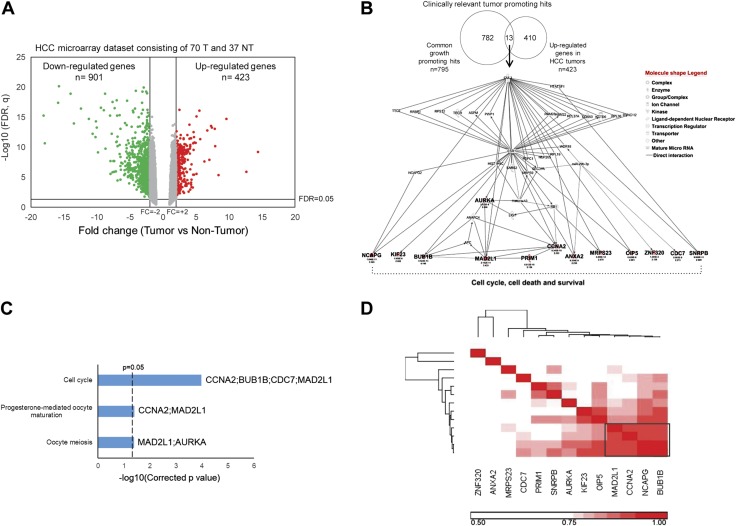
The clinically relevant targets from the CRISPR screen are enriched in the cell cycle pathway. *A*) Volcano plot showing significantly overexpressed genes (red) and underexpressed genes (green) between 70 HCC tumors and 37 nontumor samples. Significantly differentially expressed genes were indicated in the 2 upper lateral quadrants with absolute FC >2 and *q* < 0.05. *B*) Top: Venn diagram showing 13 clinically relevant targets by overlapping 795 common hits from the depletion screen with 423 significantly overexpressed genes in HCC tumors. Bottom: ingenuity pathway analysis showing all 13 clinically relevant targets (in bold) that participated in the interplay of cell cycle and cell death and survival networks. *C*) KEGG pathway enrichment analysis showing significant enrichment in cell cycle among the 13 clinically relevant targets. *D*) Pearson correlation matrix of the 13 clinically relevant targets showing a tight cluster of MAD2L1, CCNA2, NCAPG, and BUB1B (black box) in the HCC microarray dataset shown in *A*. ANXA2, annexin A2; APC, adenomatous polyposis coli; ASPM, abnormal spindle microtubule assembly; AURKA, aurora kinase A; CDC7, cell division cycle 7; CUL3, Cullin 3; DDX50, DExD-box helicase 50; DYNC1I2, dynein cytoplasmic 1 intermediate chain 2; ESR1, estrogen receptor 1; HIST1H4C, histone cluster 1 H4 family Member C; HTATSF1, HIV-1 tat specific factor 1; KIF23, kinesin family member 23; LIG1, DNA ligase 1; KCTD6, potassium channel tetramerization domain containing 6; MRPS9, mitochondrial ribosomal protein S9; NT, nontumor; NUP205, nucleoporin 205; OIP5, Opa interacting protein 5; PRIM1, DNA primase subunit 1; PSPC1, paraspeckle component 1; PWP1, PWP1 homolog, endonuclein; RB1, RB transcriptional corepressor 1; RBM, RNA binding motif protein; RPL, ribosomal protein L; RPS12, ribosomal protein S12; RRBP1, ribosome binding protein 1; SARS2, seryl-TRNA synthetase 2; SEC31A, SEC31 homolog A, COPII Coat Complex component; SNRPB, small nuclear ribonucleoprotein polypeptides B and B1; T, tumor; TECR, *trans*-2,3-enoyl-CoA reductase; TTC5, tetratricopeptide repeat domain 5; WDR18, WD repeat domain 18; ZNF320, zinc finger protein 320.

To shortlist genes that showed tumor-specific expression from the normal cells, we performed whole-genome expression microarrays on 70 HCC tumors and 37 matched nontumor samples and identified a total of 423 genes that were significantly overexpressed in the tumor samples (FDR *q* < 0.05 and FC >2) (**Fig. 2*A***). Overlapping CRISPR screens and HCC expression microarray data, we found 13 clinically relevant targets that are essential for HCC tumor cell growth and were significantly up-regulated in HCC tumors (Fig. 2*B*). The tumor-specific up-regulations of these 13 genes in HCC tumors were further confirmed in 2 independent HCC datasets [GSE14520 microarray and The Cancer Genome Atlas (TCGA) RNA-seq, **Table 1**]. Ingenuity pathway analysis revealed all the 13 genes in the cell cycle, cell death, and survival interacting networks (Fig. 2*B*), whereas KEGG pathway analysis showed cell cycle as the most significantly enriched pathway among these 13 clinically relevant targets (Fig. 2*C*). Pearson correlation revealed a prominent 4-gene cluster in which mitotic arrest deficient 2 like 1 (MAD2L1), cyclin A2 (CCNA2), and BUB1 mitotic checkpoint serine/threonine kinase B (BUB1B) were well characterized in HCC and the cell cycle (Fig. 2*D*). NCAPG was ranked as the top gene based on the *P* value from the depletion screen of both cell lines and the highest FC in HCC tumors compared with nontumors of 3 independent studies (Table 1). In addition, NCAPG was one of the most highly expressed genes among the 13 genes in a panel of 25 HCC cell lines, and it was expressed at very low levels in most adult tissues (Supplemental Fig. S2). Taken together, these data demonstrated that the tumor-specific up-regulation of NCAPG was commonly observed in HCC. Targeting NCAPG can potentially and specifically inhibit HCC tumors with few side effects on healthy tissues.

**TABLE 1:** Clinically relevant targets identified from the CRISPR cell growth screen

Rank	Clinically relevant targets	C-score	CRISPR depletion screen *P* value		HCC microarray (T/NT)		GSE14520 microarray (T/NT)		TCGA HCC RNA-seq (T/NT)	
			HCCLM3	SNU449	FC	FDR, *q*	FC	FDR, *q*	FC	FDR, *q*
1	NCAPG	17	0.001	0.040	3.5	7.6E−10	9.9	7.7E−07	20.9	1.6E−43
2	CCNA2	22	0.004	0.003	2.3	1.6E−08	7.1	7.8E−06	11.4	9.5E−36
3	AURKA	25	0.002	0.035	2.6	5.2E−08	8.6	4.5E−06	6.7	4.6E−38
4	MAD2L1	25	0.001	0.016	3.4	8.6E−10	7.1	4.4E−06	4.9	6.8E−28
5	KIF23	26	0.000	0.003	2.1	8.7E−06	3.5	2.0E−05	12.2	8.5E−32
6	BUB1B	32	0.035	0.042	4.1	4.0E−11	7.7	8.3E−06	17.0	3.4E−37
7	OIP5	32	0.034	0.012	2.1	5.5E−07	7.9	1.3E−07	8.5	6.6E−30
8	PRIM1	36	0.015	0.015	2.2	1.4E−08	7.0	3.6E−06	2.5	1.1E−12
9	CDC7	40	0.003	0.046	2.3	1.1E−07	6.8	2.3E−06	4.4	1.5E−23
10	ANXA2	48	0.025	0.035	2.2	8.6E−08	5.9	1.8E−04	2.2	1.6E−11
11	SNRPB	49	0.003	0.033	2.0	2.9E−09	1.6	1.9E−01	2.1	9.6E−19
12	ZNF320	49	0.004	0.015	2.1	1.8E−03	—	—	1.7	2.6E−03
13	MRPS23	55	0.019	0.018	2.1	2.2E−10	—	—	1.7	7.7E−18

CRISPR depletion screen *P* value is calculated based on the MAGeCK algorithm. C-score is the sum of ranking of the 13 genes based on their individual ranking according to *P* value of CRISPR screen and FC of the 3 expression datasets. C-score, combined score; NT, nontumor; T, tumor.

### Transient knockdown of NCAPG inhibits cell growth, migration, and mitochondrial gene expression

**Figure 3:**
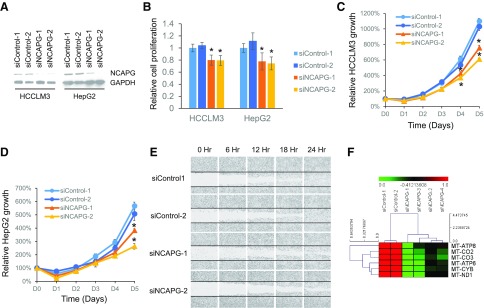
Transient knockdown of NCAPG inhibits cell growth, migration, and mitochondrial gene expression. *A*) Western blot showing the successful knockdown of NCAPG in HCCLM3 and HepG2 cells transfected with siRNAs targeting NCAPG (siNCAPG-1 and -2) compared with either control siRNAs (siControl-1 and -2). with glyceraldehyde-3-phosphate dehydrogenase (GAPDH) as endogenous control. *B*) Relative cell proliferation measured using MTS assay showing significantly reduced cell proliferation potential in cells transfected with siRNAs targeting NCAPG compared with control cells. *C*, *D*) Relative cell growth showing significantly slower cell growth in NCAPG knockdown HCCLM3 (*C*) and HepG2 (*D*) cells *vs.* control, measured using cell counting with Trypan blue exclusion assay. *E*) Wound healing assay showing reduced cell migration in NCAPG knockdown cells compared with control cells. Horizontal lines mark the margins of the initial wound. *F*) Heat map showing significant down-regulation of mitochondrial genes in the electron transport chain in NCAP knockdown cells compared with control cells. CO, cytochrome C oxidase; CYB, cytochrome B; MT, mitochondrially encoded; ND1, NADH:ubiquinone oxidoreductase core subunit 1.

To experimentally validate the CRISPR screen data, we transiently knocked down NCAPG expression by transfecting 2 sequence-independent siRNAs targeting NCAPG separately into HCCLM3 and HepG2 HCC cell lines (**Fig. 3*A***). Compared with the nontargeting controls, we observed a significant reduction in cell proliferation in NCAPG knockdown cells by measuring the cell’s ability to metabolize the tetrazolium compound [3-(4,5-dimethylthiazol-2-yl)-5-(3-carboxymethoxyphenyl)-2-(4-sulfophenyl)-2H-tetrazolium, inner salt (MTS)] (Fig. 3*B*) and cell counting with Trypan blue exclusion assays (Fig. 3*C, D*). In addition, NCAPG knockdown cells also showed significantly reduced cell migration in wound healing assays (Fig. 3*E*) and lower expression of mitochondrial genes involved in the electron transport chain and energy production (Fig. 3*F*). Therefore, we successfully validated that NCAPG is important for HCC tumor cell growth.

### Constitutive knockdown of NCAPG significantly impairs cell viability

**Figure 4:**
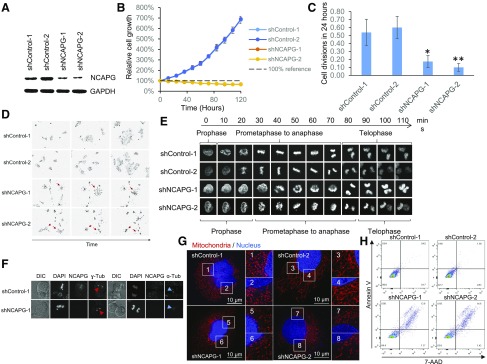
Constitutive knockdown of NCAPG significantly impairs HCC cell survival. *A*) Western blot showing the successful knockdown of NCAPG in HCCLM3 cells transduced with lentivirus expressing shRNAs targeting NCAPG (shNCAPG-1 and -2) compared with either control shRNAs (shControl-1 and -2). with GAPDH as endogenous control. *B*) Relative cell growth showing cells constitutively expressing shRNAs targeting NCAPG (shNCAPG-1 and -2) failed to proliferate *in vitro*. *C*) Mean number of cell divisions per starting cell during 24-h period (recorded using time-lapse live-cell imaging) showed significantly fewer cell divisions in constitutive NCAPG knockdown cells compared with control cells. *D*) Representative images showing cells with constitutive knockdown of NCAPG died after cell division (red arrows). Magnification value, ×10. *E*) Time-lapse confocal microscopy on H2B–enhanced green fluorescent protein-expressing HCCLM3 cells showing slowed kinetics in condensing and decondensing chromosomes in mitosis in constitutive NCAPG knockdown cells compared with control cells. Magnificaiton value, ×40. *F*) Immunofluorescence staining showed the cellular localization of DNA, NCAPG, α-tubulin, and γ-tubulin in mitosis. Constitutive NCAPG knockdown cells showed normal centrosome assembly (red arrows) and furrowing during cytokinesis (blue arrows) compared with control cells. Magnification value, ×60 *G*) Superresolution confocal microscopy images showing extensive fragmentation of mitochondrial network (region 5–8) in cells with constitutive knockdown of NCAPG compared with control cells (region 1–4), revealed through staining using MitoTracker (red) and Hoechst (blue) (Sanofi, Paris, France). Magnification value, ×120. Scale bars, 10 µm. *H*) FACS analysis showed significantly higher cell deaths in constitutive NCAPG knockdown cells, as indicated by 7-aminoactinomycin D (7-AAD)–positive cells. DIC, differential interference contrast. *P < 0.05, **P < 0.01.

To further functionally characterize NCAPG in HCC, we transduced HCC cells with lentiviruses constitutively expressing shRNAs targeting NCAPG or nontargeting controls (**Fig. 4*A***). Surprisingly, HCC cells stably expressing shRNAs against NCAPG failed to proliferate *in vitro* (Fig. 4*B*). Further examination through time-lapse live-cell imaging revealed that HCC cells with constitutive NCAPG inhibition rarely divided, and the few cells that went through cell division died soon after cell division (Fig. 4*C, D*). In the very few NCAPG knockdown cells that went through cell division, it required significantly longer time to condense and decondense the chromosome during prophase and telophase, respectively, whereas the same processes happened within minutes in control cells (Fig. 4*E*). Once the chromosome was condensed, the time duration from prometaphase to anaphase was comparable between NCAPG knockdown cells and the control cells.

NCAPG protein expression level did not show significant changes during the cell cycle (Supplemental Fig. S3). In control cells, the NCAPG protein colocalized with the chromosome at the onset of prometaphase, remained chromosome bound until anaphase, and dissociated from the chromosomes during cytokinesis, which usually overlapped with telophase. In NCAPG knockdown cells, chromosome condensation could happen in the absence of NCAPG, albeit at much lower efficiency. In these very few NCAPG knockdown cells where chromosome condensation was completed, proper bipolar assembly of centrosomes and cytokinesis furrowing were observed, suggesting the cells were able to go through normal prometaphase to anaphase. However, the daughter cells failed to decondense their chromosome even when the cells were ready to complete cytokinesis (Fig. 4*F*).

In HCC cells with constitutive NCAPG knockdown, we also observed extensive fragmentation of the mitochondrial network (Fig. 4*G*) and cell death (Fig. 4*H*). A combination of low cell division and high cell deaths associated with cell division makes it impossible to obtain a stable HCC cell line with constitutive knockdown of NCAPG.

### Conditional knockdown of NCAPG inhibited xenograft tumor growth *in vivo*

**Figure 5:**
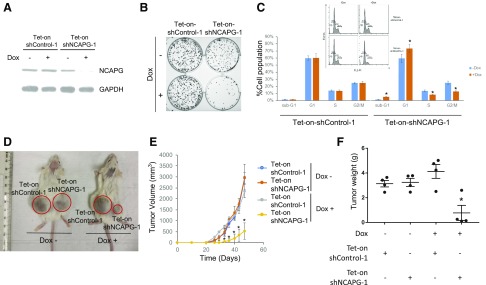
Conditional knockdown of NCAPG inhibits tumor growth *in vivo*. *A*) Western blot analysis showing the specific dox-mediated knockdown of NCAPG in cells with tetracycline-inducible shRNA against NCAPG. *B*) Colony formation assay showing dox-induced NCAPG knockdown cells formed significantly fewer colonies. *C*) Cell cycle analysis using propidium iodide staining and FACS showing a significantly reduced cell population in S and G_2_/M phase and a correspondingly higher cell population in G_1_ and sub-G_1_ phase in dox-induced NCAPG knockdown cells compared with control cells. *D*) Representative mouse xenograft imaging showing dox-induced NCAPG knockdown cells formed significantly smaller tumors. *E*) Tumor volume of mouse xenograft tumor formed by cells containing tetracycline-inducible shRNA against NCAPG or control with or without dox treatment, over time. *F*) Boxplot of final tumor weight of xenograft tumors shown in *E*. *P < 0.05.

We proceeded to generate stable cell lines with tetracycline-inducible expression shRNA targeting NCAPG (**Fig. 5*A***). Cells with dox-induced NCAPG knockdown showed significantly slower cell growth and reduced capacity to form colonies *in vitro* (Supplemental Fig. S5 and Fig. 5*B*). Consistent with the imaging data in Fig. 4, FACS analysis of the cell cycle showed significantly smaller populations of S and G_2_/M cells and a higher population of G_1_ and sub-G_1_ cells in dox-induced NCAPG knockdown cells compared with control cells (Fig. 5*C*). Furthermore, dox-induced knockdown of NCAPG resulted in slower xenograft tumor growth and significantly smaller tumors compared with the controls (Fig. 5*D–F*). Therefore, stable knockdown of NCAPG significantly impaired cell growth both *in vitro* and *in vivo*.

### High NCAPG expression in tumors is a prognostic factor for early HCC recurrence

**Figure 6:**
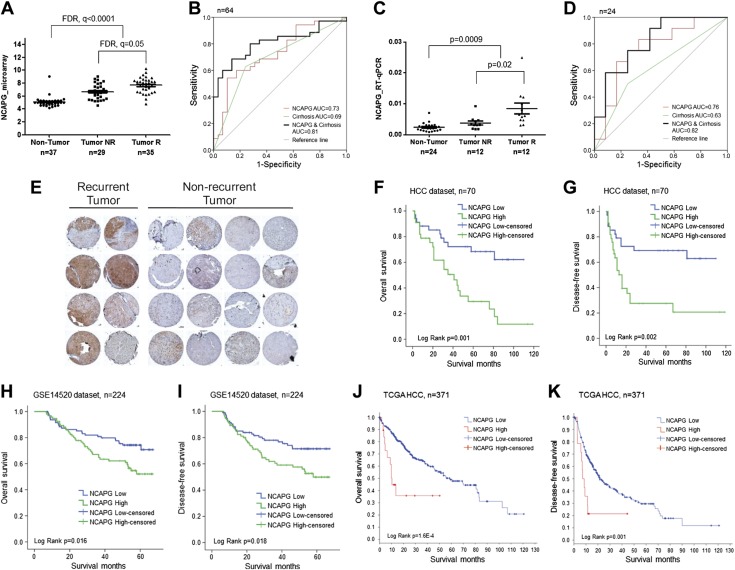
NCAPG is significantly associated with tumor recurrence postresection and poorer patient survival. *A*) NCAPG is significantly up-regulated in early recurrent tumors compared with nonrecurrent tumors in our HCC microarray dataset. *B*) ROC curve analysis shows NCAPG, cirrhosis, or NCAPG and cirrhosis in combination in discriminating recurrent and nonrecurrent HCC tumors in these 64 patients. *C*) NCAPG is validated using qRT-PCR to be significantly overexpressed in 12 independent early recurrent tumors compared with 12 independent nonrecurrent tumors. *D*) ROC curve analysis shows NCAPG, cirrhosis, or NCAPG and cirrhosis in combination in discriminating recurrent and nonrecurrent HCC tumors in these 24 independent samples. *E*) Immunohistochemistry staining showed significantly higher NCAPG levels in recurrent tumors compared with nonrecurrent tumors. *F*–*K*) High NCAPG level is significantly associated with poorer overall survival in the HCC dataset (*F*), GSE14520 dataset (*H*), and TCGA HCC dataset (*J*). High NCAPG level is significantly associated with poorer disease-free survival in the HCC dataset (*G*), GSE14520 dataset (*I*), and TCGA HCC dataset (*K*). NR, nonrecurrent; R, recurrent.

High NCAPG expression in HCC was significantly associated with α-fetoprotein–positive tumors, higher-grade tumors, and early tumor recurrence (**Table 2**). NCAPG expression was significantly higher in the 35 early recurrent tumors (patients who suffered relapses within the first 2 yr after potentially curative surgery) compared with the 29 patients in the nonrecurrent group who did not show recurrence in the 5-yr duration (**Fig. 6*A***). In this cohort of 64 patients, NCAPG transcript expression could discriminate early recurrent tumors from nonrecurrent tumors with an area under the curve (AUC) of 0.73 (*P* = 0.002). Furthermore, high NCAPG and positive liver cirrhosis improved the AUC to 0.81 (*P* = 0.000) (Fig. 6*B*). These observations were independently validated with a cohort of 24 HCC patients (consisting of 12 early recurrent and 12 nonrecurrent tumors) using qRT-PCR. NCAPG transcript level was consistently and significantly higher in the recurrent tumors (Fig. 6*C*). Similarly, NCAPG expression alone and when in combination with the presence of liver cirrhosis could discriminate early HCC recurrent disease with an AUC of 0.76 (*P* = 0.028) and 0.82 (*P* = 0.008), respectively, in this validation patient cohort (Fig. 6*D*). Another independent cohort of 32 HCC tumors was examined using immunohistochemistry for the protein expression of NCAPG. Positive NCAPG staining was prominent in 7 out of the 8 recurrent tumors and 2 out of the 24 nonrecurrent tumors studied, yielding an estimated sensitivity of 87.5% and a specificity of 91.7% (Fig. 6*E*). Moreover, high NCAPG expression is significantly associated with poorer overall survival and disease-free survival in an HCC expression dataset (*n* = 70) previously established in our laboratory (E-MEXP-84 and E-TABM-292) (Fig. 6*F, G*). This observation was confirmed in 2 larger independent datasets: the GSE14520 HCC expression dataset (*n* = 224) and the TCGA HCC RNA-seq dataset (*n* = 371) (Fig. 6*H–K*). Therefore, high NCAPG expression in HCC tumors can be explored as a potential prognostic factor for early HCC recurrence and poorer survival.

**TABLE 2:** Clinicopathologic correlation of NCAPG up-regulation in human HCC

Clinicopathological parameters	Total cases (*n* = 70)	NCAPG expression		
		High NCAPG (*n* = 35)	Low NCAPG (*n* = 35)	*P*
Gender				0.15
Male	61	28 (46%)	33 (54%)	
Female	9	7 (38%)	2 (62%)	
Age (yr)				0.63
≤60	35	19 (54%)	16 (46%)	
>60	35	16 (46%)	19 (54%)	
Etiology				0.26
HBV	57	30 (53%)	27 (47%)	
HCV	1	0 (0%)	1 (100%)	
Unknown	12	5 (42%)	7 (58%)	
Tumor Differentiation				0.04*
I and II	47	19 (40%)	28 (60%)	
III and IV	23	16 (70%)	7 (30%)	
Tumor size (cm)				0.79
≤3	20	9 (45%)	11 (55%)	
>3	50	26 (52%)	24 (48%)	
BCLC Staging				0.75
0 or A	57	29 (51%)	28 (49%)	
B, C, or D	12	5 (42%)	7 (58%)	
Unknown	1	1 (100%)	0 (0%)	
Vascular Invasion				0.80
Yes	26	14 (54%)	12 (46%)	
No	44	21 (48%)	23 (52%)	
Tumor Multifocality				0.15
Single	61	33 (54%)	28 (46%)	
Multiple	9	2 (22%)	7 (78%)	
Serum AFP (ng/ml)				0.03*
≤20	34	12 (35%)	22 (65%)	
>20	36	23 (64%)	13 (36%)	
Tumor Recurrence				0.01*
Yes	35	22 (63%)	13 (37%)	
No	29	9 (31%)	20 (69%)	
Unknown	6	4 (67%)	2 (33%)	
Liver Cirrhosis				0.34
Yes	33	19 (58%)	14 (42%)	
No	37	16 (43%)	21 (57%)	
Child-Pugh Status				0.79
A	53	27 (51%)	26 (49%)	
B	17	8 (47%)	9 (53%)	

High and low NCAPG level are determined by comparing with the median expression. *P* value is calculated from Fisher exact test. AFP, α-fetoprotein; BCLC, Barcelona Clinic liver cancer; HBV, hepatitis B virus; HCV, hepatitis C virus. *P < 0.05. The number in each cell indicates the number of cases in each sub-category, whereas the percentage within brackets indicates the percentage of cases in each category with respect to the total cases in that category.

## DISCUSSION

Conventional chemotherapeutic drugs are largely ineffective in treating HCC, and full molecular understanding of the process of hepatocarcinogenesis remains a major challenge. Although there is no single molecular or genetic alteration known presently to contribute sufficiently hepatocarcinogenesis (3, 10), high cell proliferation was the most prominent single characteristic of cancer cells and was associated with poorer survival in HCC patients (11, 12). Many upstream genetic and epigenetic modulations of core signaling pathways in HCC will eventually affect genes that play vital roles in cell division, and aberrant cell proliferation is the definitive hallmark of cancer (20). Therefore, in the absence of applicable molecular pathways to target, we have developed strategies to search for relevant therapeutic targets in the cell cycle and upstream regulators of signal pathways regulating cell proliferation. We performed genome-wide CRISPR knockout depletion screens and identified 795 genes that were essential for HCC tumor cell growth. Not surprisingly, many of these hits are involved in a number of essential signaling pathways, such as ribosome biogenesis, cell cycle, and DNA replication (Fig. 1*B*), and therefore most of these hits are not suitable therapeutic targets. To identify promising therapeutic targets that are tumor specific, we overlapped these 795 genes with 423 genes that are significantly up-regulated genes in HCC tumors. Only 13 genes were tumor specific, as evident by their significant overexpression in the HCC tumors, and they were predominantly enriched in the cell cycle pathway, demonstrating that the cell cycle is a viable source to search for novel targets for therapy (Fig. 2). Among these 13 clinically relevant targets, NCAPG was the most significantly and consistently up-regulated gene in all the 25 HCC cell lines and a combined cohort of 551 primary HCC tumors from 3 independent datasets (Supplemental Fig. S2*B* and Table 1). In comparison, NCAPG was expressed minimally in most adult normal tissues other than the testis (Supplemental Fig. S2*A*). Inhibiting NCAPG in normal hepatocytes did not significantly alter the cell morphology, growth, and viability (Supplemental Fig. S6). Therefore, inhibiting NCAPG function represents a new strategy worthy of exploring to treat HCC because it could potentially offer a high degree of tumor specificity and few side effects in other tissues with the proper delivery methods.

We experimentally validated NCAPG as a true target identified from the CRISPR screen to play an important role for HCC tumor cell growth. This is consistent with a recently published study in which Zhang *et al.* (21) also demonstrated that inhibiting NCAPG negatively affects cell growth. As a crucial component of the condensin complex (22), NCAPG binds to the chromosome when the chromatin starts to condense during the start of mitotic division and dissociates from the chromosome when it starts to unwind at telophase (Fig. 4*F*). When NCAPG was constitutively suppressed, cell division was significantly inhibited because the cancer cells take a much longer time to condense and decondense chromosome before and after nuclear division through a backup NCAPG-independent mechanism that is much less efficient. These NCAPG knockdown cells showed extensive fragmentation of the mitochondrial network and reduced expression of mitochondrial genes involved in the electron transport chain, which are typically associated with increased cell death (23) (Figs. 3*F* and 4*G*). Many cells undergo cell death soon after such abnormal cell division (Fig. 4*D, H*). Although mitotic catasphophe and mitochondial fragmentation are both reported to be associated with cell death (24), we cannot yet prove whether NCAPG knockdown–mediated mitotic catasphophe directly or indirectly caused mitochondrial fragmentation and cell death. Nonetheless, targeting NCAPG in HCC tumor cells will likely achieve the compound effect of fewer and slower cell divisions, a fragmented mitochondrial network, and cell death, resulting in significantly impaired tumor cell growth *in vitro* and *in vivo*. Interestingly, NCAPG up-regulation is also observed in most of the 34 cancer types in the TCGA database, suggesting the possibility of targeting NCAPG as a generic cell cycle target to treat a broad range of cancers (Supplemental Fig. S4).

High NCAPG expression is significantly associated with tumor recurrence. Currently, surgical resection and liver transplantation offer the best potential for treating HCC (25–27). Unfortunately, more than half of the patients who undergo resection eventually succumb to tumor recurrence, with a median recurrence time of <2 yr (28). Here we demonstrated that NCAPG transcript level in combination with liver cirrhosis could discriminate early recurrent tumors from nonrecurrent tumors with an AUC of >0.80 in 2 independent datasets, whereas NCAPG protein level can correctly identify 7 out of the 8 early recurrent tumors (Fig. 6*B, D, E*). Moreover, high NCAPG expression is associated with significantly poorer patient disease-free and overall survival in multiple datasets (Fig. 6*F–K*). Thus, NCAPG can be utilized as a prognostic biomarker to predict the risk of recurrence in HCC patients after liver resection.

In summary, from a genome-wide functional knockout screen, we identified and characterized NCAPG as a clinically relevant target essential for HCC tumor cell growth. Targeting NCAPG may represent a novel strategy of treating HCC tumors, and the prognostic biomarker potential of NCAPG in predicting early HCC recurrence should also be evaluated in a larger patient cohort to aid the clinical management of HCC.

## Supplementary Material

This article includes supplemental data. Please visit *http://www.fasebj.org* to obtain this information.

## Abbreviations

AUC: area under the curve
BUB1B: BUB1 mitotic checkpoint serine/threonine kinase B
CCNA2: cyclin A2
CRISPR: clustered regularly interspaced short palindromic repeats
dox: doxycycline
FACS: fluorescence-activated cell sorting
FC: fold change
FDR: false discovery rate
gRNA: guide RNA
HCC: hepatocellular carcinoma
KEGG: Kyoto Encyclopedia of Genes and Genomes
MAD2L1: mitotic arrest deficient 2 like 1
MAGeCK: Model-based Analysis of Genome-wide CRISPR/Cas9 Knockout
MOI: multiplicity of infection
NCAPG: non–structural maintenance of chromosomes condensin I complex subunit G
qRT-PCR: quantitative RT-PCR
RNA-seq: RNA sequencing
shRNA: small homologous RNA
siRNA: small interfering RNA
TCGA: The Cancer Genome Atlas
