# Supplementary material for: Genome-wide CRISPR knockout screens identify NCAPG as an essential oncogene for hepatocellular carcinoma tumor growth
Source: FASEB J. 2019 Apr 25;33(8):8759–70. doi: 10.1096/fj.201802213RR (PMC6662966; doi:10.1096/fj.201802213RR)
Supplement: Supplementary file 1 [file fj.201802213RR.sd1.pdf]

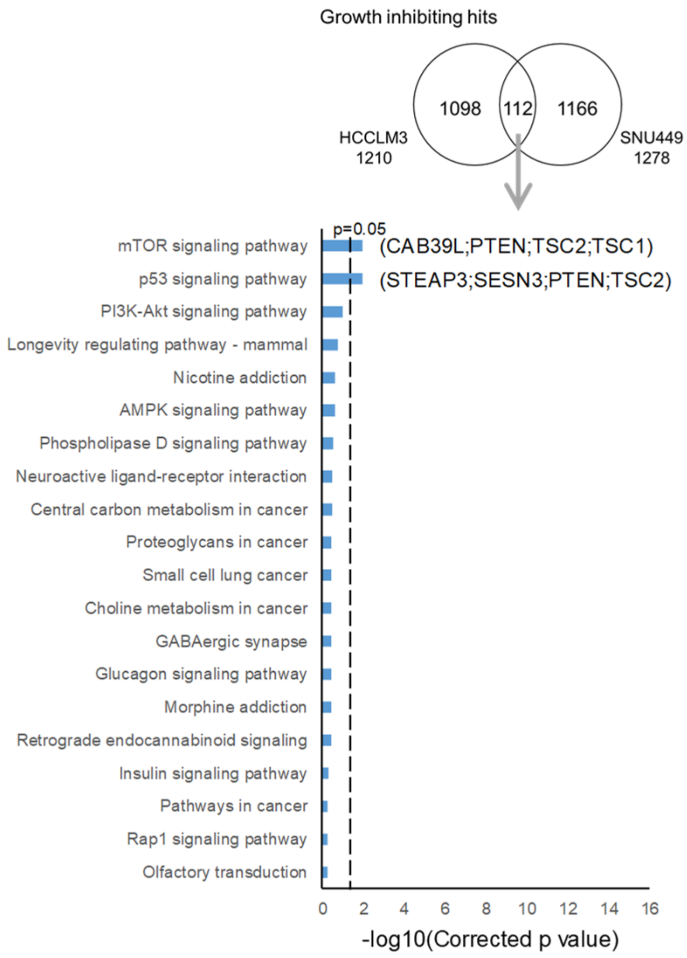

Supplementary Fig S1. Enriched hits from the CRISPR growth screen were significantly enriched in negative regulator of cell growth. *Top:* Venn diagram showing a total of 112 hits were commonly enriched in both HCCLM3 and SNU449 cell lines at the end of the screen. *Bottom*: KEGG pathway analysis showing significant enrichment in mTOR signaling and p53 signaling pathways which function as negative regulators of cell growth, amongst these 112 commonly enriched targets. The x-axis shows the p value from hypergeometric test adjusted by the multiple test correction. The dash line indicates a threshold of corrected p value=0.05.

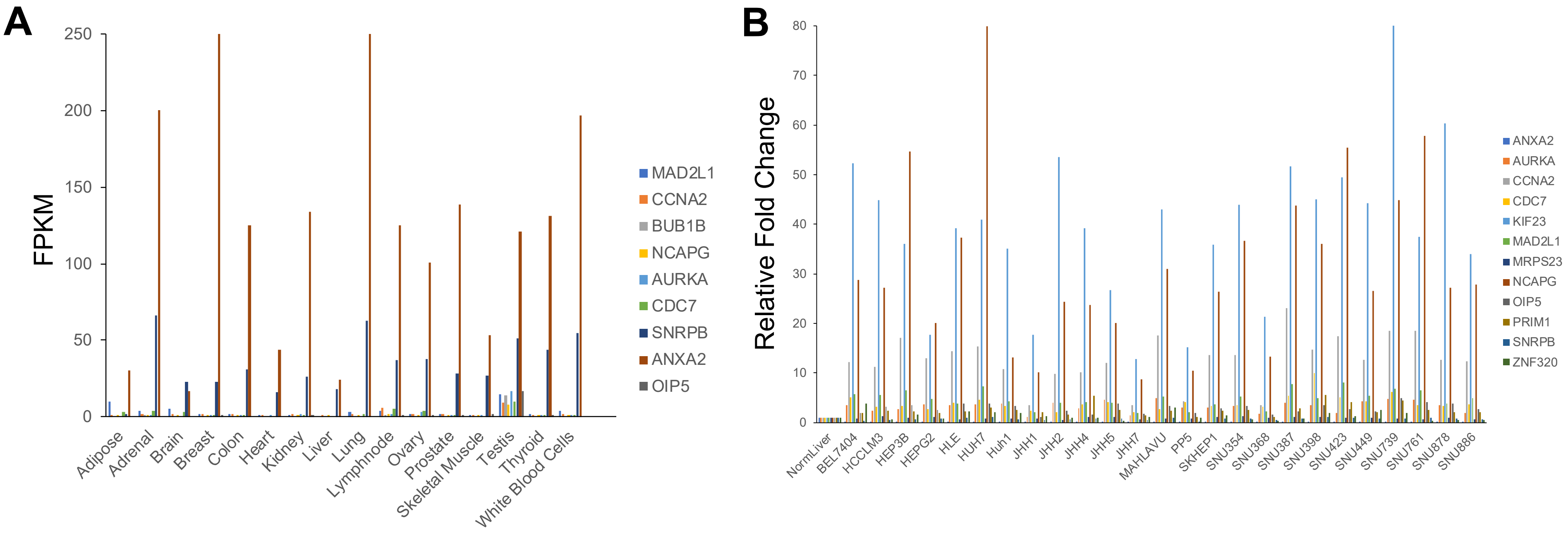

Supplementary Fig. S2 (A) Normalized transcript expression (FPKM) of the clinically relevant targets in human tissue panels measured RNA-seq from Illumina BodyMap project. **(B)** Relative fold change of clinically relevant target transcript expression in HCC cell lines normalized against Normal Liver.

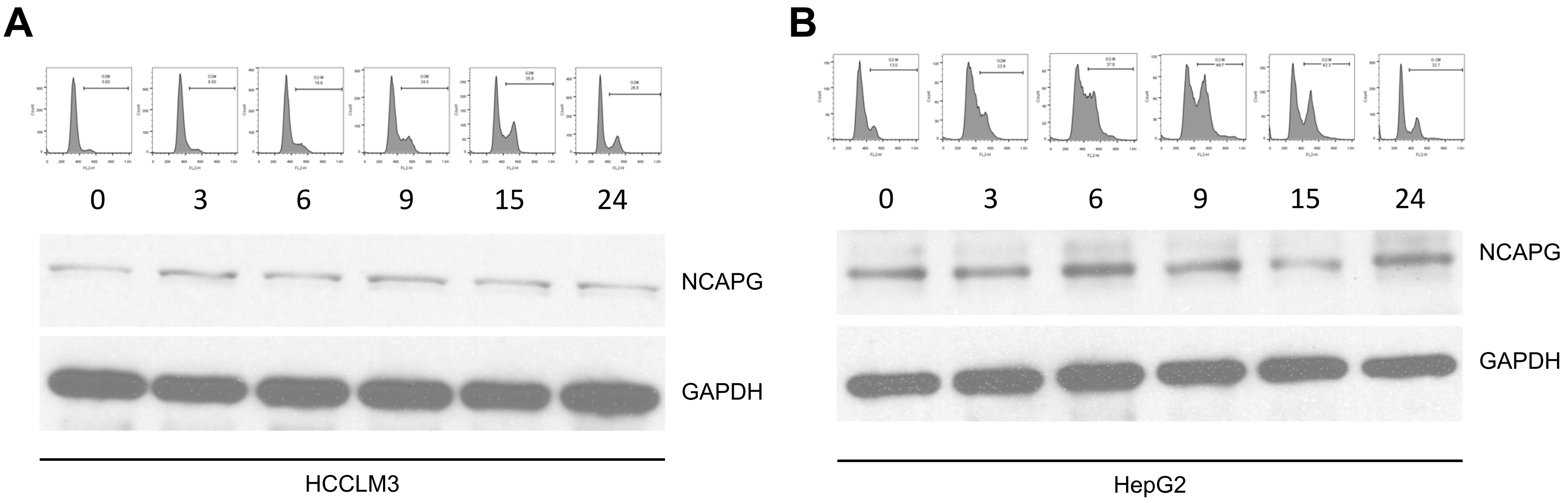

Supplementary Fig. S3 NCAPG expression is not cell cycle regulated. HCCLM3 cells **(A)** and HepG2 cells **(B)** were arrested at G1 phase using double thymidine method and released into normal growth media. *Upper panel:* Cells were harvested at various time points 0-24 hours post release and cell cycle profile were analyzed using PI staining and FACS analysis. *Bottom panel:* NCAPG protein expression was analyzed in the same cells using western blot analysis with GAPDH as endogenous control.

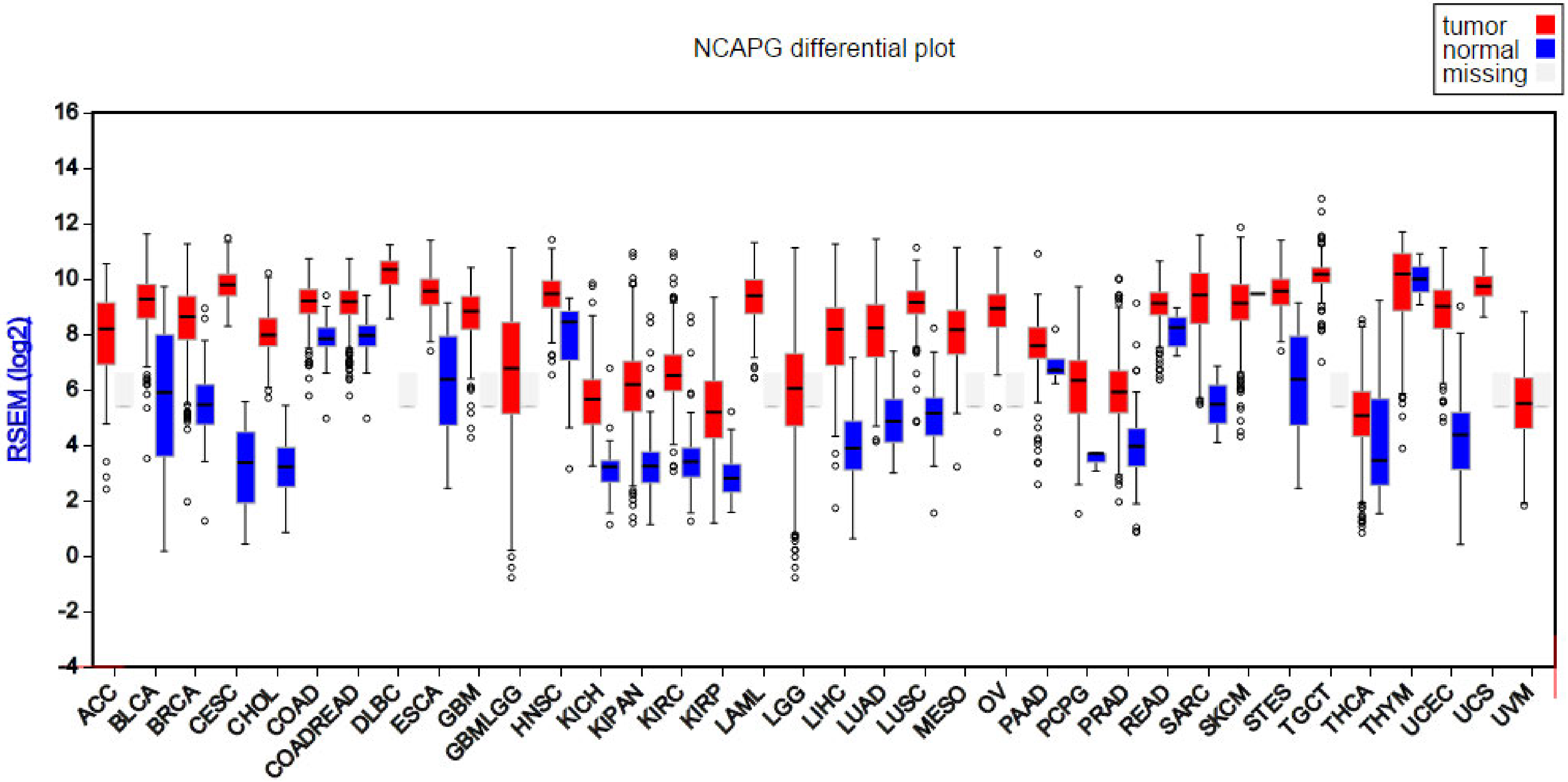

Supplementary Fig. S4 NCAPG is found to be frequently up-regulated in most cancers. Boxplot of NCAPG transcript measured using RNA-seq in 36 different cancer types from TCGA datasets showed significant up-regulation of NCAPG in tumor (red boxes) versus matching normal samples (blue boxes) in majority of cancers.

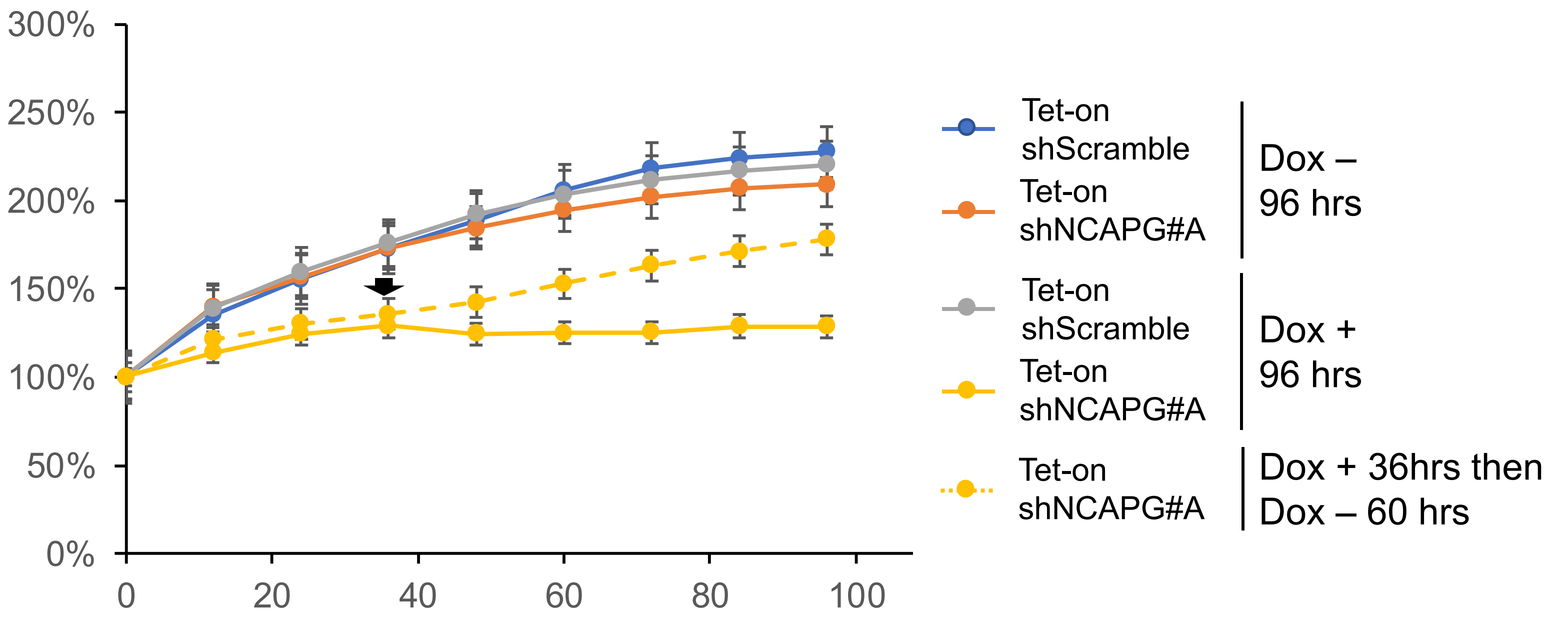

Supplementary Fig. S5 Dox-inducible inhibition of NCAPG resulted in significant inhibition on cell proliferation in vitro when Dox was present for the entire 96 hrs (Yellow line). When Dox was withdrawn after initial 36 hrs (black arrow) to reverse the inhibition on NCAPG, we observed corresponding rescue in cell proliferation (Yellow dotted line)

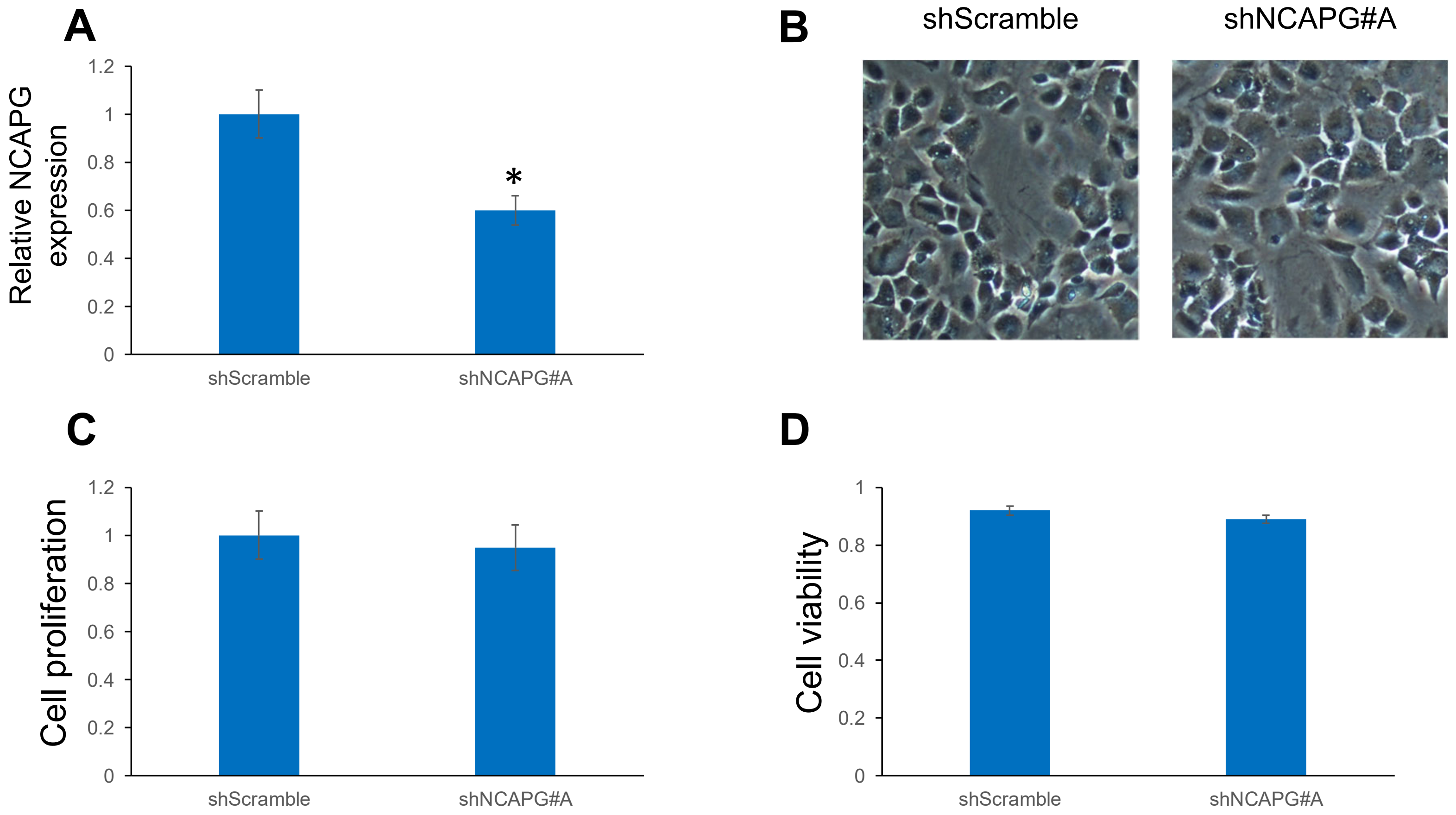

Supplementary Fig. S6 Knock-down of NCAPG in primary normal hepatocytes (A) did not significantly change the cell morphology(B), cell proliferation (C) or cell viability(D), measured using cell counting with trypan blue exclusion.

Supplementary Table S1: Information on primers, siRNAs, shRNAs and antibodies used in the study

	Name	Sequence 5'-3'/Cat. No
Primers for library construction	v2Adaptor_F	AATGGACTATCATATGCTTACCGTAACTTGAAAGTATTTCG
	v2Adaptor_R	TCTACTATTCTTTCCCCTGCACTGTtgtgggcgatgtgcgctctg
	F8N	AATGATACGGCGACCACCGAGATCTACACTCTTTCCCTACACGACGCTCTTCCGATCTNNNNNNNNtcttgtggaaaggacgaaacaccg
	R02	CAAGCAGAAGACGGCATACGAGATACACGATCGTGACTGGAGTTCAGACGTGTGCTCTTCCGATCTatTCTACTATTCTTTCCCCTGCACTGT
	R03	CAAGCAGAAGACGGCATACGAGATCGCGCGGTGTGACTGGAGTTCAGACGTGTGCTCTTCCGATCTgatTCTACTATTCTTTCCCCTGCACTGT
	R05	CAAGCAGAAGACGGCATACGAGATCGTTACCAGTGACTGGAGTTCAGACGTGTGCTCTTCCGATCTtcgatTCTACTATTCTTTCCCCTGCACTGT
	R07	CAAGCAGAAGACGGCATACGAGATAACGCATTGTGACTGGAGTTCAGACGTGTGCTCTTCCGATCTgatcgatTCTACTATTCTTTCCCCTGCACTGT
	R08	CAAGCAGAAGACGGCATACGAGATACAGGTATGTGACTGGAGTTCAGACGTGTGCTCTTCCGATCTcgatcgatTCTACTATTCTTTCCCCTGCACTGT
	R10	CAAGCAGAAGACGGCATACGAGATAACAATGGGTGACTGGAGTTCAGACGTGTGCTCTTCCGATCTtTCTACTATTCTTTCCCCTGCACTGT
RT-qPCR primers	GAPDH-F	CATTTCCTGGTATGACAACGA
	GAPDH-R	CTTCCTCTTGTGCTCTTGCT
	NCAPG-F	AAGAAAGAACTCAAGATGGCTG
	NCAPG-R	AGCATCATTCTTCTCTATGTGG
siRNAs and shRNAs	siControl1	Sigma: SIRNA UNIV NEGATIVE CONTROL #1, SIC001
	siControl2	Sigma: SIRNA UNIV NEGATIVE CONTROL #2, SIC002
	siNCAPG#1	UCUACUCAGCUAAAGACUA(dTdT)
	siNCAPG#2	GAUUCAAAUCCAGAAGUUA(dTdT)
	siNCAPG#3	GACUAAUCAGGAAUGCUUU(dTdT)
	siNCAPG#4	CAGGAUUCUUUCUCGUCUU(dTdT)
	shScramble	GTGTAACACGTCTATACGCCCA
	shControl#B	GCAAGCTGACCCTGAAGTTCAT
	shNCAPG#A	AAGCAGGACTAATCAGGAATG
	shNCAPG#B	AATCCCAATATCCCTGGTTTC
Antibodies	NCAPG	Santa cruz: sc-101014
	NCAPG	Sigma: HPA039613
	GAPDH	Santa cruz: sc-25778
	TUBB	Sigma: T9026
	TUBG1	Sigma: T6557

Supplementary Table S2: Performance statistics of the CRISPR screen

Sample	gRNAs from Gecko v2 library	Unique gRNAs in library	Unique gRNAs in the actual screens	% coverage in the screen
Total gRNA	122,411	119,461^	119,386	99.94%
Genes	19,050	18,981	18,981	100%
miRNAs	1,864	1,823	1,823	100%
Controls	1,000	1,000	1,000	100%

The human GECKO V2 library contains a total of 122,411 gRNAs targeting a total of 19,050 genes among which 119,461 gRNAs targeting 18,981 genes are unique. Our final screens recorded a total of 119,386 gRNAs (99.94% of the library) targeting 18,981 genes (100% of the library).

Term	Overlap	P-value	Adjusted P-value	Z-score	Combined Score	Genes
Ribosome_Homo sapiens_hsa03010	74/137	0	0	-1.75	156	RPL4;RPL5;RPL30;RPL3;MRPS16;RPL32;MRPS14;RPL34;MRPS12;RPLP0;MRPS10;MRPL36;MRPL34;RPL10A;RPL9;MRPL33;RPS4X;MRPL3;RPS14;RPL7A;RPS17;RPS16;RPS19;RPL18A;RPS1 ;RPL35;RPLP2;RPL38;MRPL9;RPL37;RPL39;RPL21;RPS8;RPL23;RPS5;RPS6;MRPS18A;RPL13A;MRPS2;MRPS21;RPS3A;MRPS6;RPL37A;RPL24;RPL27;RPL26;RPL28;RPS10-
Spliceosome_Homo sapiens_hsa03040	31/134	8E-10	7.4E-08	-1.77	29	SF3B5;TCERG1;DDX46;DDX23;DDX42;HNRNPU;PRPF19;SNRPD1;U2AF2;TRA2B;DHX15;SF3A3;PRPF38B;SF3A1;NCBP1;BUD31;PRPF40A;LSM4;LSM3;CHERP;LSM7;SNRNP40;SNW1;NHP2
Ribosome biogenesis in eukaryotes_Homo sapiens_hsa03008	24/89	6E-09	3.4E-07	-1.93	29	UTP15;POP5;NOP58;POP7;IMP3;NVL;WDR3;HEATR1;IMP4;GNL2;SPATA5;AK6;PWP2;UTP18;GTPBP4;WDR43;EFTUD1;FBL;NHP2L1;EIF6;NOB1;XRN2;RIOK1;RPP3
Aminoacyl-tRNA biosynthesis_Homo sapiens_hsa00970	19/66	1E-07	3.9E-06	-2.05	26	RARS2;YARS;DARS;PARS2;MARS2;EPRS;YARS2;NARS;SARS2;LARS;MARS;NARS2;IARS2;HARS;IARS;FARSA;CARS2;AARS;PST
RNA transport_Homo sapiens_hsa03013	31/172	1E-07	4E-06	-1.75	22	POP5;POP7;SUMO2;SMN2;SAP18;NUP43;SMN1;EIF2B1;RAE1;RPP38;EIF2B4;UPF1;PRMT5;UBE2I;EIF2B2;NUP155;NCBP1;EIF1AX;RANGAP1;SNUPN;NUP93;CLNS1A;RPP21;EIF3G;GEMIN
Cell cycle_Homo sapiens_hsa04110	26/124	1E-07	3.9E-06	-1.63	20	PCNA;MCM7;CCNH;CUL1;BUB1B;PKMYT1;CDC23;ORC6;CDC45;MYC;CHEK1;BUB3;SKP1;ZBTB17;PLK1;CDC7;RBX1;MAD2L2;CCNA2;CDC16;CDK2;ANAPC4;MCM5;ANAPC5;MAD2L1;MC
RNA polymerase_Homo sapiens_hsa03020	13/32	5E-07	1E-05	-1.46	17	POLR2J3;POLR2J2;POLR3A;POLR3C;POLR2C;POLR3E;POLR2D;POLR2E;POLR1E;POLR3H;POLR2H;POLR3K;POLR2L
Proteasome_Homo sapiens_hsa03050	15/44	4E-07	9.4E-06	-1.43	17	PSMD12;PSMA7;PSMB6;PSMD8;PSMA6;PSMB7;PSMA3;PSMB5;PSMD7;PSMC3;PSMB3;PSMC1;PSMC2;PSMB1;PSMD1
Huntington's disease_Homo sapiens_hsa05016	29/193	9E-06	0.00018	-1.70	15	NDUFB9;NDUFB10;COX4I1;ATP5A1;NDUFB3;NDUFB2;AP2A1;ATP5B;POLR2C;POLR2D;AP2S1;POLR2E;POLR2H;POLR2L;POLR2J3;POLR2J2;TBP;NDUFA6;NDUFA2;NDUFA1;NDUFC1;ATP5
Pyrimidine metabolism_Homo sapiens_hsa00240	20/105	1E-05	0.00021	-1.54	13	POLR2J3;PRIM2;DUT;RRM1;POLR2J2;PRIM1;NUDT2;POLD3;POLR3A;POLR3C;POLR2C;POLR3E;POLR2D;CMPK1;POLR2E;POLR1E;POLR3H;POLR2H;POLR3K;POLR2L
Oxidative phosphorylation_Homo sapiens_hsa00190	21/133	8E-05	0.00134	-1.57	10	NDUFB9;NDUFA6;NDUFB10;ATP6AP1;COX4I1;ATP5A1;NDUFB3;NDUFA2;NDUFB2;NDUFA1;NDUFC1;ATP5F1;ATP5B;NDUFS8;NDUFS5;UQCRC1;ATP6V1B2;NDUFS2;NDUFS1;ATP6V0D1;
mRNA surveillance pathway_Homo sapiens_hsa03015	16/91	0.0002	0.0027	-1.39	8	UPF1;CPSF4;NCBP1;SSU72;PPP2CA;NUDT21;PABPN1;FIP1L1;WDR82;PCF11;SAP18;SYMPK;ETF1;RNPS1;BCL2L2-PABPN1;PELC
DNA replication_Homo sapiens_hsa03030	10/36	0.0001	0.00209	-1.28	8	RFC5;POLD3;PRIM2;PCNA;MCM7;RPA3;PRIM1;RPA1;MCM5;MCM2
Epstein-Barr virus infection_Homo sapiens_hsa05169	24/202	0.0012	0.01447	-1.60	7	POLR2J3;PSMD12;POLR2J2;TBP;PSMD8;CCNA2;POLR3A;PSMD7;SNW1;PSMC3;POLR3C;PSMC1;MYC;POLR2C;PSMC2;POLR3E;CDK2;POLR2D;POLR2E;PSMD1;POLR3H;POLR2H;POLR3K;
Alzheimer's disease_Homo sapiens_hsa05010	21/168	0.0014	0.01532	-1.47	6	NDUFB9;NDUFA6;NDUFB10;COX4I1;ATP5A1;NDUFB3;NDUFA2;NDUFB2;NDUFA1;ATP2A2;NDUFC1;ATP5F1;HSD17B10;ATP5B;NDUFS8;NDUFS5;UQCRC1;NDUFS2;NDUFS1;NAE1;GAPD
Nucleotide excision repair_Homo sapiens_hsa03420	10/47	0.0009	0.01111	-1.26	6	RFC5;DDB1;POLD3;GTF2H2C;PCNA;CCNH;RPA3;RPA1;XPC;RBX1
Parkinson's disease_Homo sapiens_hsa05012	18/142	0.0026	0.02704	-1.39	5	NDUFB9;NDUFA6;NDUFB10;COX4I1;ATP5A1;NDUFB3;NDUFA2;NDUFB2;NDUFA1;NDUFC1;UBE2G2;ATP5F1;ATP5B;NDUFS8;NDUFS5;UQCRC1;NDUFS2;NDUFS
Oocyte meiosis_Homo sapiens_hsa04114	16/123	0.0035	0.03444	-1.43	5	PLK1;CUL1;SPDYE6;PKMYT1;RBX1;AURKA;MAD2L2;PPP2CA;CDC23;SPDYE2B;CDC16;CDK2;ANAPC4;ANAPC5;MAD2L1;SKP1
Purine metabolism_Homo sapiens_hsa00230	20/176	0.0049	0.04267	-1.33	4	POLR2J3;PRIM2;RRM1;POLR2J2;PRIM1;NUDT2;AK6;POLD3;POLR3A;POLR3C;GUK1;POLR2C;POLR3E;POLR2D;POLR2E;POLR1E;POLR3H;POLR2H;POLR3K;POLR2L
Ubiquitin mediated proteolysis_Homo sapiens_hsa04120	17/137	0.0042	0.03829	-1.22	4	DET1;UBE2I;SMURF1;CUL1;UBE2G2;PRPF19;RBX1;DDB1;CDC23;UBE2Q1;CDC16;UBA3;UBA2;ANAPC4;ANAPC5;UBE2M;SKP
Progesterone-mediated oocyte maturation_Homo sapiens_hsa04914	12/98	0.0162	0.11883	-1.32	3	MAD2L2;CCNA2;CDC23;SPDYE2B;CDC16;PLK1;CDK2;ANAPC4;SPDYE6;ANAPC5;PKMYT1;MAD2L1
RNA degradation_Homo sapiens_hsa03018	10/77	0.0194	0.13546	-1.10	2	EXOSC6;LSM7;CNOT7;EXOSC10;XRN2;CNOT3;EXOSC8;LSM4;DCPS;LSM3
Non-alcoholic fatty liver disease (NAFLD)_Homo sapiens_hsa04932	15/151	0.0367	0.22521	-1.27	2	NDUFB9;NDUFA6;NDUFB10;COX4I1;NDUFB3;NDUFA2;NDUFB2;NDUFA1;NDUFC1;RXRA;NDUFS8;NDUFS5;UQCRC1;NDUFS2;NDUFS1
Cytosolic DNA-sensing pathway_Homo sapiens_hsa04623	Aug-64	0.0411	0.23986	-0.67	1	POLR3A;POLR3C;POLR3E;POLR2E;POLR3H;POLR2H;POLR3K;POLR2L
N-Glycan biosynthesis_Homo sapiens_hsa00510	Jul-49	0.0313	0.20294	-0.58	1	DPAGT1;RPN2;DAD1;RPN1;ALG2;DOLK;STT3B
Vibrio cholerae infection_Homo sapiens_hsa05110	Jun-51	0.0884	0.49913	-0.62	0	SEC61A1;ATP6AP1;ATP6V1B2;ATP6V0D1;ATP6V0C;ACTE
Basal transcription factors_Homo sapiens_hsa03022	May-45	0.1346	0.71387	-0.29	0	TBP;GTF2H2C;TAF13;CCNH;TAF2
Synaptic vesicle cycle_Homo sapiens_hsa04721	Jun-63	0.1725	0.86252	-0.41	0	NSF;AP2S1;ATP6V1B2;AP2A1;ATP6V0D1;ATP6V0C
Metabolic pathways_Homo sapiens_hsa01100	76/1239	0.3001	1	-1.02	0	DPAGT1;COX4I1;CPOX;PIGW;EPRS;GMPPB;UROD;GUK1;SEPHS1;POLG;MECR;TPI1;ATP6AP1;ALG2;NDUFC1;ATP5F1;NDUFS8;DAD1;NDUFS5;HMBS;UQCRC1;ATP6V1B2;CMPK1;NDUFS2; OLR1E;NDUFS1;ATP6V0D1;ALDOA;GAPDH;ATP6V0C;CERS1;CERS2;NDUFB9;PRIM2;FH;MVK;GALT;NDUFB10;RPN2;PRIM1;ATP5A1;NDUFB3;RPN1;NDUFB2;AK6;HSD17B10;PGS1;POLD3;
Protein processing in endoplasmic reticulum_Homo sapiens_hsa04141	12/169	0.2824	1	-0.91	0	UFD1L;SEC61A1;VCP;RPN2;DAD1;RPN1;CUL1;HYOU1;STT3B;UBE2G2;RBX1;SKP1
Thyroid hormone signaling pathway_Homo sapiens_hsa04919	8/118	0.3805	1	-0.68	0	MED14;RXRA;MED30;MYC;ATP2A2;ACTB;MTOR;MED17
HIF-1 signaling pathway_Homo sapiens_hsa04066	7/103	0.3926	1	-0.63	0	EGLN2;TFRC;RPS6;ALDOA;GAPDH;MTOR;RBX1
TGF-beta signaling pathway_Homo sapiens_hsa04350	Jun-84	0.3665	1	-0.63	0	PPP2CA;SMURF1;MYC;CUL1;RBX1;SKP1
Viral carcinogenesis_Homo sapiens_hsa05203	11/205	0.6365	1	-0.48	0	DDB1;CCNA2;SNW1;GTF2H2C;HIST2H4B;TBP;PSMC1;CHEK1;CDK2;HIST1H4J;ATP6V0D1
Systemic lupus erythematosus_Homo sapiens_hsa05322	8/135	0.5219	1	-0.45	0	HIST2H2AA4;HIST2H4B;SNRPD1;H2AFX;HIST1H4J;HIST2H3D;HIST2H3C;SNRPB
Epithelial cell signaling in Helicobacter pylori infection_Homo sapiens_hsa05120	May-68	0.3666	1	-0.40	0	ATP6AP1;ATP6V1B2;PTPN11;ATP6V0D1;ATP6V0C
Phagosome_Homo sapiens_hsa04145	8/154	0.6642	1	-0.24	0	SEC61A1;ATP6AP1;TFRC;TUBB;ATP6V1B2;ATP6V0D1;ATP6V0C;ACTE
Biosynthesis of amino acids_Homo sapiens_hsa01230	May-74	0.4324	1	-0.23	0	TPI1;MAT2A;ACO2;ALDOA;GAPDH
Herpes simplex infection_Homo sapiens_hsa05168	9/185	0.7382	1	-0.16	0	TBP;TAF13;CDK2;SRSF2;CUL1;SRSF3;PTPN11;SRSF9;SKP1
AMPK signaling pathway_Homo sapiens_hsa04152	6/124	0.7194	1	-0.13	0	RAB2A;CCNA2;PPP2CA;SCD;EEF2;MTOR
Endocytosis_Homo sapiens_hsa04144	12/259	0.8116	1	0.03	0	TSG101;TFRC;CAPZB;SMURF1;AP2S1;CHMP2A;AP2A1;ARPC4;WASH1;SNF8;CHMP6;VPS28
HTLV-I infection_Homo sapiens_hsa05166	12/258	0.8078	1	0.06	0	POLD3;CDC23;PCNA;TBP;MYC;CDC16;CHEK1;BUB1B;ANAPC4;ANAPC5;BUB3;MAD2L1
Renal cell carcinoma_Homo sapiens_hsa05211	Apr-66	0.5349	1	0.09	0	FH;EGLN2;PTPN11;RBX1
Pathogenic Escherichia coli infection_Homo sapiens_hsa05130	Apr-55	0.4023	1	0.11	0	TUBB;NCL;ARPC4;ACTB
Fanconi anemia pathway_Homo sapiens_hsa03460	Apr-53	0.3772	1	0.12	0	RAD51;RPA3;RPA1;TELO2
Porphyrin and chlorophyll metabolism_Homo sapiens_hsa00860	Apr-42	0.2404	1	0.13	0	UROD;HMBS;CPOX;EPRS
Shigellosis_Homo sapiens_hsa05131	Apr-65	0.5234	1	0.17	0	MAD2L2;ARPC4;PFN1;ACTB
Small cell lung cancer_Homo sapiens_hsa05222	Apr-86	0.7307	1	0.20	0	RXRA;MAX;MYC;CDK2
Peroxisome_Homo sapiens_hsa04146	Apr-83	0.7058	1	0.21	0	MVK;PMVK;SOD2;SOD1
Wnt signaling pathway_Homo sapiens_hsa04310	6/142	0.825	1	0.25	0	CSNK1A1;MYC;RUVBL1;CUL1;RBX1;SKP1
Carbon metabolism_Homo sapiens_hsa01200	5/113	0.7784	1	0.28	0	FH;TPI1;ACO2;ALDOA;GAPDH
Rheumatoid arthritis_Homo sapiens_hsa05323	Apr-90	0.7614	1	0.29	0	ATP6AP1;ATP6V1B2;ATP6V0D1;ATP6V0C
Hepatitis B_Homo sapiens_hsa05161	6/146	0.8435	1	0.31	0	CCNA2;DDB1;PCNA;ATP6AP1;MYC;CDK2
mTOR signaling pathway_Homo sapiens_hsa04150	Mar-60	0.6754	1	0.39	0	RPS6;MLST8;MTOR
Alcoholism_Homo sapiens_hsa05034	7/179	0.888	1	0.40	0	HIST2H2AA4;HIST2H4B;HAT1;H2AFX;HIST1H4J;HIST2H3D;HIST2H3C
Longevity regulating pathway - multiple species_Homo sapiens_hsa04213	Mar-64	0.7154	1	0.41	0	SOD2;MTOR;SOD1
Transcriptional misregulation in cancer_Homo sapiens_hsa05202	7/180	0.8912	1	0.44	0	ZBTB17;RXRA;MAX;MYC;HIST2H3D;HIST2H3C;KDM6A
Sphingolipid metabolism_Homo sapiens_hsa00600	Mar-47	0.5179	1	0.65	0	KDSR;CERS1;CERS2
Glycolysis / Gluconeogenesis_Homo sapiens_hsa00010	Mar-67	0.7429	1	0.66	0	TPI1;ALDOA;GAPDH
Adipocytokine signaling pathway_Homo sapiens_hsa04920	Mar-70	0.7682	1	0.74	0	RXRA;PTPN11;MTOR
Fatty acid metabolism_Homo sapiens_hsa01212	Mar-48	0.5314	1	0.74	0	MECR;SCD;TECR
Fructose and mannose metabolism_Homo sapiens_hsa00051	Mar-32	0.2973	1	0.82	0	GMPPB;TPI1;ALDOA
Bacterial invasion of epithelial cells_Homo sapiens_hsa05100	Mar-78	0.8259	1	0.87	0	MAD2L2;ARPC4;ACTB
Circadian rhythm_Homo sapiens_hsa04710	Mar-30	0.2671	1	0.94	0	CUL1;RBX1;SKP1
Cardiac muscle contraction_Homo sapiens_hsa04260	Mar-78	0.8259	1	0.98	0	COX4I1;UQCRC1;ATP2A2
Salmonella infection_Homo sapiens_hsa05132	Mar-86	0.8709	1	0.98	0	ARPC4;PFN1;ACTB
Collecting duct acid secretion_Homo sapiens_hsa04966	Mar-27	0.2226	1	1.04	0	ATP6V1B2;ATP6V0D1;ATP6V0C
Influenza A_Homo sapiens_hsa05164	5/175	0.9716	1	1.11	0	CPSF4;PABPN1;BCL2L2-PABPN1;RAE1;ACTB
Sphingolipid signaling pathway_Homo sapiens_hsa04071	3/120	0.9675	1	1.15	0	PPP2CA;CERS1;CERS2
Lysosome_Homo sapiens_hsa04142	3/123	0.9714	1	1.19	0	ATP6AP1;ATP6V0D1;ATP6V0C
Thyroid cancer_Homo sapiens_hsa05216	Feb-29	0.5099	1	1.22	0	RXRA;MYC
Chronic myeloid leukemia_Homo sapiens_hsa05220	Feb-73	0.9214	1	1.34	0	MYC;PTPN11
Hepatitis C_Homo sapiens_hsa05160	3/133	0.9815	1	1.34	0	PPP2CA;RXRA;EIF3E
FoxO signaling pathway_Homo sapiens_hsa04068	3/133	0.9815	1	1.35	0	PLK1;CDK2;SOD2
Central carbon metabolism in cancer_Homo sapiens_hsa05230	Feb-67	0.8969	1	1.35	0	MYC;MTOR
ErbB signaling pathway_Homo sapiens_hsa04012	Feb-87	0.959	1	1.37	0	MYC;MTOR
Tight junction_Homo sapiens_hsa04530	3/139	0.9858	1	1.42	0	PPP2CA;SYMPK;ACTB
Prostate cancer_Homo sapiens_hsa05215	Feb-89	0.9627	1	1.43	0	CDK2;MTOR
Longevity regulating pathway - mammal_Homo sapiens_hsa04211	Feb-94	0.9706	1	1.45	0	SOD2;MTOR
Vasopressin-regulated water reabsorption_Homo sapiens_hsa04962	Feb-44	0.7236	1	1.45	0	NSF;DCTN5
Proteoglycans in cancer_Homo sapiens_hsa05205	5/203	0.9903	1	1.46	0	MYC;RPS6;PTPN11;ACTB;MTOR
Endocrine and other factor-regulated calcium reabsorption_Homo sapiens_hsa04	Feb-47	0.7555	1	1.48	0	AP2S1;AP2A1
Inositol phosphate metabolism_Homo sapiens_hsa00562	Feb-71	0.9139	1	1.51	0	TPI1;CDIPT
Dilated cardiomyopathy_Homo sapiens_hsa05414	Feb-90	0.9644	1	1.52	0	ATP2A2;ACTB
Tuberculosis_Homo sapiens_hsa05152	4/178	0.9911	1	1.53	0	ATP6AP1;NFYC;ATP6V0D1;ATP6V0C
Acute myeloid leukemia_Homo sapiens_hsa05221	Feb-57	0.8398	1	1.55	0	MYC;MTOR
Glycerophospholipid metabolism_Homo sapiens_hsa00564	Feb-95	0.972	1	1.57	0	PGS1;CDIPT
PPAR signaling pathway_Homo sapiens_hsa03320	Feb-69	0.9058	1	1.58	0	RXRA;SCD
Notch signaling pathway_Homo sapiens_hsa04330	Feb-48	0.7654	1	1.58	0	JAG1;SNW1
Insulin resistance_Homo sapiens_hsa04931	2/109	0.9858	1	1.58	0	PTPN11;MTOR
Amino sugar and nucleotide sugar metabolism_Homo sapiens_hsa00520	Feb-48	0.7654	1	1.60	0	GMPPB;GALT
Hippo signaling pathway_Homo sapiens_hsa04390	3/153	0.9924	1	1.61	0	PPP2CA;MYC;ACTB
Pancreatic secretion_Homo sapiens_hsa04972	Feb-96	0.9733	1	1.61	0	CTRB1;ATP2A2
p53 signaling pathway_Homo sapiens_hsa04115	Feb-69	0.9058	1	1.62	0	CDK2;CHEK1
TNF signaling pathway_Homo sapiens_hsa04668	2/110	0.9865	1	1.64	0	JAG1;DNM1L
Hypertrophic cardiomyopathy (HCM)_Homo sapiens_hsa05410	Feb-83	0.9505	1	1.69	0	ATP2A2;ACTB
Arrhythmogenic right ventricular cardiomyopathy (ARVC)_Homo sapiens_hsa0541	Feb-74	0.9249	1	1.70	0	ATP2A2;ACTB
Jak-STAT signaling pathway_Homo sapiens_hsa04630	3/158	0.994	1	1.70	0	MYC;PTPN11;MTOR
Glutathione metabolism_Homo sapiens_hsa00480	Feb-52	0.8016	1	1.72	0	RRM1;GPX4
Viral myocarditis_Homo sapiens_hsa05416	Feb-59	0.8531	1	1.74	0	ACTB;EIF4G1
Leukocyte transendothelial migration_Homo sapiens_hsa04670	2/118	0.9909	1	1.76	0	PTPN11;ACTB
Measles_Homo sapiens_hsa05162	2/136	0.9963	1	1.88	0	CDK2;GNB2L1
Insulin signaling pathway_Homo sapiens_hsa04910	2/139	0.9968	1	1.89	0	RPS6;MTOR
Fatty acid elongation_Homo sapiens_hsa00062	Feb-25	0.4372	1	1.91	0	MECR;TECR
Apoptosis_Homo sapiens_hsa04210	2/140	0.997	1	1.95	0	AIFM1;ACTB
Base excision repair_Homo sapiens_hsa03410	Feb-33	0.5761	1	1.96	0	POLD3;PCNA
Adrenergic signaling in cardiomyocytes_Homo sapiens_hsa04261	2/148	0.998	1	2.01	0	PPP2CA;ATP2A2
Phospholipase D signaling pathway_Homo sapiens_hsa04072	2/144	0.9975	1	2.03	0	PTPN11;MTOR
PI3K-Akt signaling pathway_Homo sapiens_hsa04151	8/341	0.999	1	2.03	0	PPP2CA;RXRA;MYC;RPS6;CDK2;MLST8;THEM4;MTOR
Citrate cycle (TCA cycle)_Homo sapiens_hsa00020	Feb-30	0.527	1	2.04	0	FH;ACO2
Biosynthesis of unsaturated fatty acids_Homo sapiens_hsa01040	Feb-23	0.3988	1	2.05	0	SCD;TECR
Regulation of actin cytoskeleton_Homo sapiens_hsa04810	3/214	0.9996	1	2.09	0	ARPC4;PFN1;ACTB
Oxytocin signaling pathway_Homo sapiens_hsa04921	2/158	0.9988	1	2.11	0	EEF2;ACTB
MicroRNAs in cancer_Homo sapiens_hsa05206	4/297	1	1	2.15	0	UBE2I;MYC;KIF23;MTOR
Calcium signaling pathway_Homo sapiens_hsa04020	2/180	0.9996	1	2.15	0	P2RX2;ATP2A2
Olfactory transduction_Homo sapiens_hsa04740	4/415	1	1	2.18	0	OR4F17;OR4F16;OR11H12;OR4F29
Rap1 signaling pathway_Homo sapiens_hsa04015	2/211	0.9999	1	2.21	0	PFN1;ACTB
Natural killer cell mediated cytotoxicity_Homo sapiens_hsa04650	1/135	0.9995	1	2.25	0	PTPN11
Fc gamma R-mediated phagocytosis_Homo sapiens_hsa04666	Jan-93	0.995	1	2.25	0	ARPC4
Pathways in cancer_Homo sapiens_hsa05200	9/397	0.9997	1	2.27	0	FH;EGLN2;RAD51;RXRA;MAX;MYC;CDK2;MTOR;RBX1
Platelet activation_Homo sapiens_hsa04611	1/122	0.999	1	2.28	0	ACTB
Neurotrophin signaling pathway_Homo sapiens_hsa04722	1/120	0.9989	1	2.28	0	PTPN11
Glucagon signaling pathway_Homo sapiens_hsa04922	1/101	0.9968	1	2.29	0	PPP4C
Neuroactive ligand-receptor interaction_Homo sapiens_hsa04080	1/277	1	1	2.29	0	P2RX2
Signaling pathways regulating pluripotency of stem cells_Homo sapiens_hsa04550	1/142	0.9997	1	2.29	0	MYC
Taste transduction_Homo sapiens_hsa04742	Jan-83	0.9912	1	2.30	0	P2RX2
Prolactin signaling pathway_Homo sapiens_hsa04917	Jan-72	0.9836	1	2.33	0	GALT
Phosphatidylinositol signaling system_Homo sapiens_hsa04070	Jan-98	0.9962	1	2.33	0	CDIPT
Chagas disease (American trypanosomiasis)_Homo sapiens_hsa05142	1/104	0.9973	1	2.33	0	PPP2CA
Choline metabolism in cancer_Homo sapiens_hsa05231	1/101	0.9968	1	2.34	0	MTOR
Focal adhesion_Homo sapiens_hsa04510	1/202	1	1	2.35	0	ACTB
MAPK signaling pathway_Homo sapiens_hsa04010	2/255	1	1	2.36	0	MAX;MYC
Hematopoietic cell lineage_Homo sapiens_hsa04640	Jan-88	0.9934	1	2.36	0	TFRC
Chemical carcinogenesis_Homo sapiens_hsa05204	Jan-82	0.9907	1	2.37	0	SULT1A4
GABAergic synapse_Homo sapiens_hsa04727	Jan-88	0.9934	1	2.37	0	NSF
Dopaminergic synapse_Homo sapiens_hsa04728	1/129	0.9994	1	2.38	0	PPP2CA
NF-kappa B signaling pathway_Homo sapiens_hsa04064	Jan-93	0.995	1	2.38	0	UBE2I
Gap junction_Homo sapiens_hsa04540	Jan-88	0.9934	1	2.39	0	TUBB
cGMP-PKG signaling pathway_Homo sapiens_hsa04022	1/167	0.9999	1	2.40	0	ATP2A2
Gastric acid secretion_Homo sapiens_hsa04971	Jan-74	0.9854	1	2.42	0	ACTB
cAMP signaling pathway_Homo sapiens_hsa04024	1/199	1	1	2.42	0	ATP2A2
Adherens junction_Homo sapiens_hsa04520	Jan-74	0.9854	1	2.43	0	ACTB
Ras signaling pathway_Homo sapiens_hsa04014	1/227	1	1	2.44	0	PTPN11
Long-term depression_Homo sapiens_hsa04730	Jan-60	0.9677	1	2.46	0	PPP2CA
Bile secretion_Homo sapiens_hsa04976	Jan-71	0.9827	1	2.48	0	RXRA
Pancreatic cancer_Homo sapiens_hsa05212	Jan-66	0.977	1	2.53	0	RAD51
Antigen processing and presentation_Homo sapiens_hsa04612	Jan-77	0.9876	1	2.56	0	NFYC
Protein digestion and absorption_Homo sapiens_hsa04974	Jan-90	0.9941	1	2.59	0	CTRB1
Endometrial cancer_Homo sapiens_hsa05213	Jan-52	0.9492	1	2.59	0	MYC
Colorectal cancer_Homo sapiens_hsa05210	Jan-62	0.9711	1	2.60	0	MYC
Glioma_Homo sapiens_hsa05214	Jan-65	0.9756	1	2.62	0	MTOR
Non-small cell lung cancer_Homo sapiens_hsa05223	Jan-56	0.9595	1	2.66	0	RXRA
Legionellosis_Homo sapiens_hsa05134	Jan-55	0.9571	1	2.67	0	VCP
Amyotrophic lateral sclerosis (ALS)_Homo sapiens_hsa05014	Jan-51	0.9463	1	2.89	0	SOD1
Type II diabetes mellitus_Homo sapiens_hsa04930	Jan-48	0.9364	1	3.00	0	MTOR
Hedgehog signaling pathway_Homo sapiens_hsa04340	Jan-50	0.9432	1	3.01	0	CSNK1A1
Bladder cancer_Homo sapiens_hsa05219	Jan-41	0.9056	1	3.02	0	MYC
Valine, leucine and isoleucine degradation_Homo sapiens_hsa00280	Jan-48	0.9364	1	3.16	0	HSD17B10
Galactose metabolism_Homo sapiens_hsa00052	Jan-30	0.8246	1	3.27	0	GALT
Pentose phosphate pathway_Homo sapiens_hsa00030	Jan-29	0.8144	1	3.31	0	ALDOA
Prion diseases_Homo sapiens_hsa05020	Jan-35	0.8676	1	3.35	0	SOD1
Cysteine and methionine metabolism_Homo sapiens_hsa00270	Jan-45	0.9246	1	3.43	0	MAT2A
Glyoxylate and dicarboxylate metabolism_Homo sapiens_hsa00630	Jan-28	0.8037	1	3.78	0	ACO2
Pyruvate metabolism_Homo sapiens_hsa00620	Jan-40	0.9001	1	3.79	0	FH
Glycosylphosphatidylinositol(GPI)-anchor biosynthesis_Homo sapiens_hsa00563	Jan-25	0.7676	1	3.96	0	PIGW
2-Oxocarboxylic acid metabolism_Homo sapiens_hsa01210	Jan-17	0.6357	1	4.08	0	ACO2
Histidine metabolism_Homo sapiens_hsa00340	Jan-24	0.7542	1	4.37	0	C9ORF41
Pantothenate and CoA biosynthesis_Homo sapiens_hsa00770	Jan-18	0.6556	1	4.44	0	AASDHPPT
Protein export_Homo sapiens_hsa03060	Mar-23	0.1662	0.85533	0.96	0	SEC61A1;SRP68;SRP9
Homologous recombination_Homo sapiens_hsa03440	Apr-29	0.1025	0.56041	0.53	0	POLD3;RAD51;RPA3;RPA1
Mismatch repair_Homo sapiens_hsa03430	May-23	0.0163	0.11883	0.33	-1	RFC5;POLD3;PCNA;RPA3;RPA1
Terpenoid backbone biosynthesis_Homo sapiens_hsa00900	May-22	0.014	0.11133	0.36	-1	MVK;FNTB;PMVK;PDSS2;DHDDS
Selenocompound metabolism_Homo sapiens_hsa00450	Apr-17	0.0248	0.16725	1.24	-2	MARS2;MARS;SEPHS1;PSTK
Ubiquinone and other terpenoid-quinone biosynthesis_Homo sapiens_hsa00130	3-Nov	0.0373	0.22521	1.76	-3	COQ2;COQ6;COQ5
Sulfur relay system_Homo sapiens_hsa04122	4-Oct	0.0056	0.04681	1.80	-6	CTU2;TRMU;NFS1;MOCS3
